# Quad-Channel In Vivo Photoacoustic Multiplexing Using Tunable Gold Nanorods

**DOI:** 10.1021/acsnano.5c22519

**Published:** 2026-05-07

**Authors:** Anamik Jhunjhunwala, Myeongsoo Kim, Huyju Mun, Brooke Chambliss, Jinhwan Kim, Stanislav Y. Emelianov

**Affiliations:** † Wallace H. Coulter Department of Biomedical Engineering, 1372 Georgia Institute of Technology and Emory University School of Medicine, Atlanta, Georgia 30332, United States; ‡ Petit Institute for Bioengineering and Bioscience, Georgia Institute of Technology, Atlanta, Georgia 30332, United States; § Department of Biomedical Engineering, 8789 University of California Davis, Davis, California 95616, United States; ∥ Department of Surgery, University of California Davis, Sacramento, California 95616, United States; ⊥ School of Electrical & Computer Engineering, Georgia Institute of Technology, Atlanta, Georgia 30332, United States

**Keywords:** photoacoustic imaging, multiplexing, gold nanorods, silica coating, spectral unmixing, in vivo molecular imaging, contrast agents

## Abstract

Photoacoustic (PA) imaging combines the molecular specificity of optical absorption with the resolution and depth of ultrasound, enabling noninvasive molecular imaging in vivo. However, robust and accurate PA multiplexing is hindered by the broad spectra and poor photostability of existing contrast agents, and interference from endogenous absorbers. Here, we demonstrate four-channel PA multiplexing using PEGylated silica-coated gold nanorods with narrow, spectrally distinct plasmon resonances spanning the near-infrared window. A reproducible three-step seed-mediated synthesis yields monodisperse, spectrally tunable gold nanorods. Co-hybrid silica encapsulation with PEGylation enhances photostability and standardizes both particle dimensions and surface chemistries across the panel. With only a commercial OPO laser and a straightforward non-negative least-squares algorithm, we achieve accurate signal separation across a full-factorial set of in vitro and in vivo models, maintaining mean absolute errors below 4.3%. We further show that the nanorod library remains clearly distinguishable in the presence of endogenous oxyhemoglobin and deoxyhemoglobin and can be unmixed using VisualSonics native spectral deconvolution. This platform doubles existing PA multiplexing capabilities to four exogenous channels without requiring multimodal imaging, complex laser setups, or complicated computational pipelines. This work establishes a framework for future applications in noninvasive biomarker mapping, multicellular therapies, and spatially resolved diagnostics.

## Graphical abstract

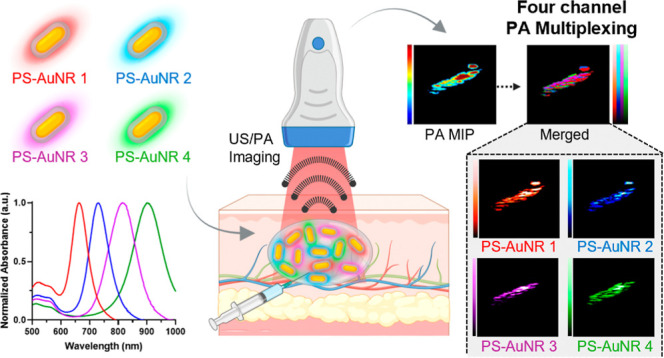

## Introduction

Molecular imaging has been of particular interest for understanding complex biological systems by enabling the noninvasive longitudinal visualization of biochemical processes, molecular interactions, and functional alterations deep within living tissue.^1,2^ Several molecular imaging modalities such as positron emission tomography (PET), magnetic resonance imaging (MRI), and optical imaging have advanced the field, however, each comes with inherent limitations. PET offers exceptional sensitivity but is hampered by low spatial resolution and the use of ionizing radiation.^3–6^ MRI provides high soft tissue contrast but lacks molecular specificity and cannot perform real-time or longitudinal monitoring.^7^ Optical imaging achieves high molecular sensitivity but is severely limited by poor resolution and shallow depth penetration.^8–10^ Ultrasound and photoacoustic imaging (US/PA) address many of these limitations, offering a combination of nonionizing radiation, molecular specificity, real-time imaging, high spatial resolution, and significant depth penetration.^11^ By integrating the acoustic resolution of US imaging with the molecular sensitivity of optical imaging, US/PA emerges as a hybrid modality, well suited for advancing molecular imaging in complex biological systems such as the tumor microenvironment.^12^ A key next step in molecular imaging is the capability to multiplex, wherein several spectrally distinct targets are detected simultaneously, providing a composite view of separate biological molecules or events.^13–16^ Unlike multiomics approaches that require ex vivo sampling and extended processing, multiplexed PA imaging offers the potential to achieve these measurements in vivo, in real time, and noninvasively. This capability is pivotal for emerging biomedical challenges that demand concurrent read-outs, such as mapping spatial cancer heterogeneity and tracking dynamic interactions of cell populations in multicellular therapies, while preserving the native physiological context.^14,17,18^

PA imaging leverages the principle of the photoacoustic effect, where pulsed laser light is absorbed by chromophores within tissue, leading to localized heating, thermoelastic expansion, and the generation of broadband ultrasound waves.^19,20^ These pressure waves propagate through tissue and are detected by ultrasound transducers, allowing for the reconstruction of high-resolution images that reveal the optical and molecular properties of the tissue.^21,22^ By leveraging the near-infrared (NIR, ∼650 to 1700 nm) optical window, where biological tissues exhibit minimal absorption and scattering, PA imaging achieves a balance of molecular sensitivity and imaging depth.^23,24^ Normally PA imaging relies on the use of endogenous molecules, such as hemoglobin and melanin, which naturally absorb light and are used for mapping oxygen saturation levels, identifying hypoxic tumor regions, and monitoring vascular dynamics.^25^ For instance, hemoglobin’s varying absorption based on oxygenation enables the mapping of hypoxic tumor regions, while melanin’s strong PA absorption makes it effective for imaging melanoma. However, for multiplexing, endogenous contrast agents are inherently limited.^20,23,26–28^ Their spectra are broad with significant crosstalk. Lacking target specificity, they are restricted to particular molecules and are unable to distinguish between closely related molecular species.^28–30^ They are subject to background noise from surrounding tissues, reducing their effectiveness in heterogeneous and dynamic environments.^31,32^ These challenges underscore the need for exogenous contrast agents that have spectral signatures different from endogenous tissue and can be tailored to target specific molecular biomarkers, enabling enhanced sensitivity, specificity, and multiplexing capabilities in PA imaging.

Effective PA multiplex contrast agents will meet specific, stringent criteria to ensure high sensitivity, resolution, and specificity.^25^ (1) Each agent must possess spectrally distinct absorption peaks with minimal overlap, allowing for clear differentiation of multiple agents within a single image.^33^ Minimizing crosstalk between signals is critical to ensure accurate quantification of individual targets, particularly in complex biological environments. (2) Additionally, they should operate within the NIR optical window, where tissue absorption and scattering are minimized, to achieve sufficient tissue penetration depth.^34^ (3) Furthermore, contrast agents must exhibit optical properties that are distinguishable from endogenous chromophores to ensure that detected signals originate primarily from the intended exogenous agents. (4) Beyond spectral properties, ideal PA contrast agents should demonstrate high photostability to maintain consistent signal intensity during prolonged imaging sessions or longitudinal imaging applications. (5) The contrast agents must exhibit biocompatibility to ensure safety in vivo.^35,36^ (6) Another crucial attribute is ease of functionalization, which allows these agents to be precisely tailored for targeting specific biomarkers. Despite significant advancements, many existing PA contrast agents face challenges in multiplexing applications, exhibiting broad overlapping spectra, insufficient photostability, or limited biocompatibility in physiological environments.^37^ This complicates spectral unmixing and limits accuracy about their local concentrations. Lastly, effective multiplexing also depends on computational algorithms capable of resolving overlapping spectral signatures and mitigating background noise.^38^ The development of contrast agents which meet these criteria paves the way for significant advancements in multiplex PA imaging.

Among exogenous PA contrast agents, gold nanorods (AuNRs) have emerged as one of the most promising contrast agents for PA multiplexing due to their intense and narrow absorption peaks, high photothermal conversion efficiency, and strong optical absorption in the NIR region, where tissue absorption and scattering are minimized.^39–42^ Their plasmon resonances can be tuned across the NIR window, enabling them to serve as spectrally distinct channels for simultaneous imaging of multiple targets.^43^ To further improve performance, silica coating of AuNRs is employed which enhances photostability, colloidal stability, and PA signal 1, 3, 4, 5 ^35,44–46^ . Furthermore, the silica surface allows for the facile conjugation of targeting ligands expanding AuNR utility for targeted molecular imaging and theranostic applications.^46,47^ Previous studies, including our own, have utilized silica-coated AuNRs (S-AuNRs) for PA multiplexing applications, demonstrating their potential for identifying multiple cell types in tissue-mimicking phantoms.^48–50^ Additionally, laboratories have explored spectrally discrete dye-based probes for PA multiplexing.^28,51–53^ However, in vivo PA multiplexing has been predominantly confined to two-agent demonstrations due to the broad absorption profiles of current probes and significant spectral overlap when another probe is introduced.^54–56^ While some studies have attempted to extend the palette with additional spectrally distinct dyes, these contrast agents typically have weak PA absorption and poor photostability, which diminishes their practical effectiveness.^52,53,57^ Moreover, many published demonstrations rely on qualitative images; they employ ad-hoc or unvalidated spectral-unmixing routines and rarely report quantitative accuracy relative to known ground-truth compositions.^58–64^ Thus, the advance in the present study is not simply the use of AuNRs for PA imaging, but the development of a practical four-channel exogenous-agent multiplexing framework based on a tunable AuNR library with matched physicochemical properties.

Building on this foundation, we developed four spectrally distinct S-AuNR contrast agents optimized for the NIR optical window that address the spectral overlap and stability limitations of earlier PA probes. We utilized a four-step seed-mediated synthesis, where step 1 produces monodisperse spherical gold seeds, step 2 converts these seeds into miniature AuNRs, step 3 tunes their aspect ratio giving spectrally unique AuNRs with narrow plasmon peaks, and step 4 silica-coats them.^65,66^ This results in S-AuNR subpopulations with narrow, well-separated plasmon peaks across the NIR window together with matching morphologies and surface chemistries. Subsequent PEGylation suppresses nonspecific aggregation and protein corona formation, yielding a library of PS-AuNRs that behave uniformly in biological environments.^67^ A non-negative least-squares unmixing algorithm was applied to spectroscopic PA images to evaluate multiplexing across a panel of single, binary, ternary, quaternary, and boundary condition mixtures in both clear and hemoglobin-rich backgrounds. In vivo multiplexing performance was further assessed in a subcutaneous murine model, including comparison against the native VisualSonics spectral deconvolution workflow and explicit testing of robustness to endogenous oxyhemoglobin and deoxyhemoglobin background. Together, these tuned probes and the accompanying multiplexing pipeline provide four channel PA multiplexing with high fidelity, expanding the toolkit for reliable identification of multiple molecular targets in heterogeneous and dynamic biological systems.

## Results

### Rational Design and Computational Optimization of Tunable AuNRs

AuNRs are typically synthesized using either seedless or seed-mediated methods (Figure S1). Among these, seed-mediated approaches are generally favored due to their superior reproducibility, nanoparticle homogeneity, and precise control over size distributions. Seed-mediated synthesis initially involves reducing gold ions to form extremely small spherical gold seed particles, which subsequently grow asymmetrically into nanorods in the presence of surfactant templates and precisely controlled reaction conditions. Seedless methods integrate nucleation and growth into a single step, simplifying the procedure but frequently resulting in heterogeneous populations, reduced reproducibility, and higher failure rates compared to seed-mediated methods. Leveraging the reliability and precision of seed-mediated strategies, we developed a modified three-step nanorod synthesis strategy to improve size uniformity and batch reproducibility, achieving fine control over aspect ratios and the optical tuning necessary for multiplexed PA imaging (Figure 1A). Our three-step approach began with the controlled generation of ultrasmall spherical gold seeds, followed by their anisotropic growth into miniature intermediate nanorods. These small rods serve as uniform, precisely sized templates for the final growth step, in which the rod length and aspect ratio are finely tuned by manipulating silver ion concentration, seed concentration, and pH conditions (Figure S1). This structured synthetic approach enables the consistent production of AuNR populations with tightly controlled geometries, precisely tuned optical resonances, and improved batch consistency, yielding optical properties optimized specifically for multiplexed PA imaging within the near-infrared-I (NIR-I, ∼650 to 1000 nm).

**Figure 1:**
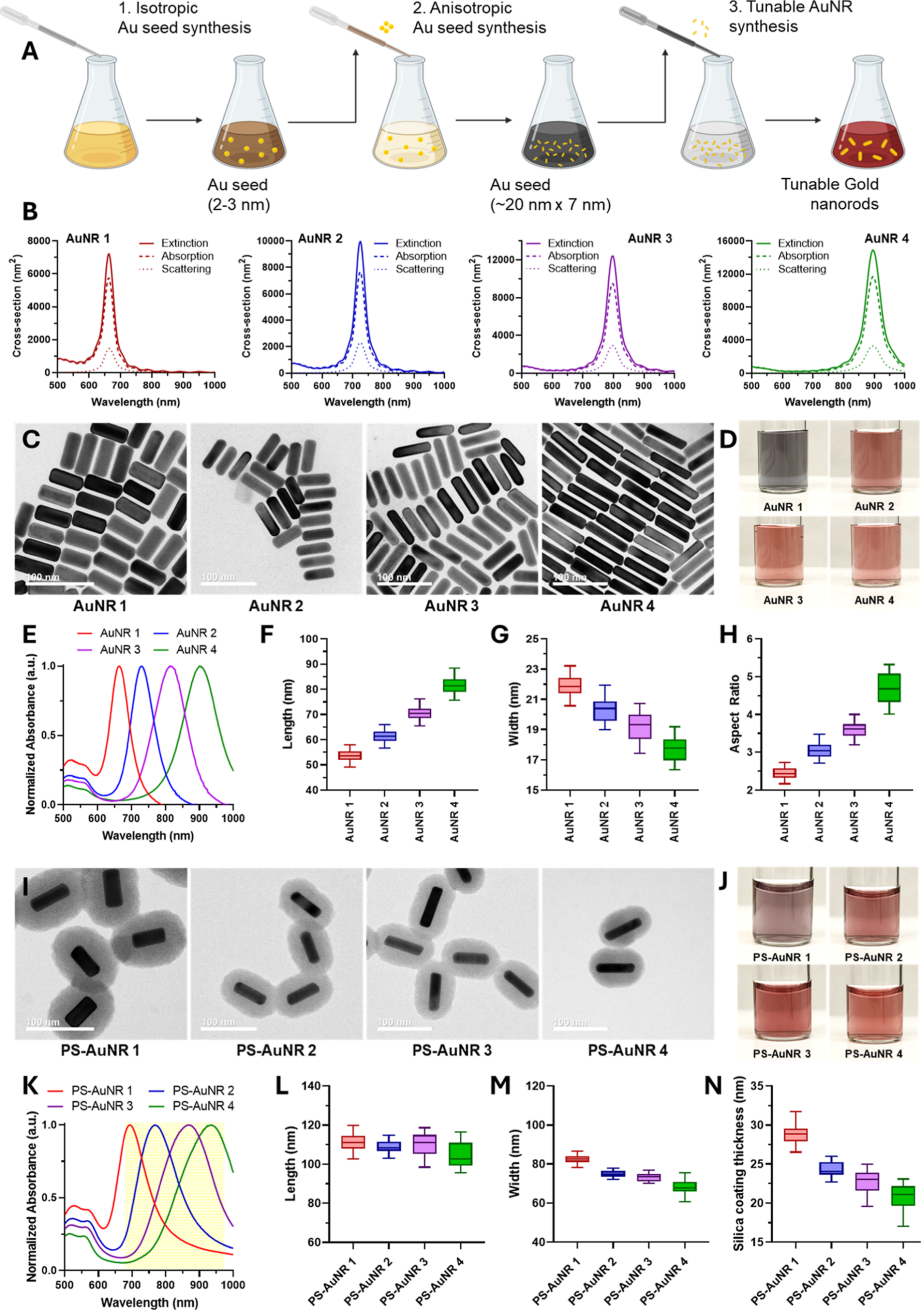
Synthesis and characterization of a spectrally distinct PS-AuNR panel for PA multiplexing. (A). Schematic illustration of the three-step tunable AuNR synthesis protocol. Step 1 yields isotropic gold seeds; Step 2 produces uniform, miniature AuNRs from these seeds; and Step 3 enables precise aspect ratio tuning. (B). FDTD simulations of extinction, absorption, and scattering cross sections for AuNR1–4. (C). TEM images of AuNR1–4 show highly monodisperse particles (Scale bar, 100 nm). (D). Colorimetric photographs of aqueous dispersions of the four nanorod formulations showing distinct solution colors. (E). UV–Vis–NIR absorbance spectra of AuNR1–4, demonstrating four well-separated longitudinal plasmon resonance peaks. (F). TEM measurements of nanorod length (*n* = 100 per group), showing progressive elongation from AuNR1–4. (G). Corresponding width measurements, revealing a decrease in diameter across the series. (H). Calculated aspect ratios with values increasing progressively from AuNR1–4 explaining progressive redshifts observed in Panel E. (I). TEM images of PEGylated, silica-coated gold nanorods (PS-AuNR1–4), showing uniform silica coating and homogeneous rod morphology (Scale bar, 100 nm). (J). Colorimetric photographs of PS-AuNR1–4 in aqueous solution, each displaying unique coloration. (K). UV–Vis–NIR absorbance spectra of PS-AuNR1–4, showing red-shifted longitudinal plasmon peaks relative to uncoated AuNRs, consistent with silica coating. (L). PS-AuNR1–4 lengths from TEM analysis (*n* = 50 per group), showing comparable rod lengths across all four formulations. (M). Corresponding width measurements (*n* = 50 per group), also showing consistent sizing after silica coating. (N). Silica shell thickness measurements for PS-AuNR1–4, demonstrating a controlled decrease in coating thickness across the series, enabling consistent nanoparticle dimensions with spectrally distinguishable AuNR cores.

To identify optimal AuNR geometries for PA multiplexing, we conducted finite-difference time-domain (FDTD) computational simulations. Four separable nanorod geometries (AuNR1–AuNR4) were selected, and optical cross sections, extinction, absorption, and scattering, were computed to determine their suitability. Simulations indicated strong and discrete extinction peaks positioned clearly within the NIR-I window (Figure 1B). Importantly, the simulations predicted minimal spectral overlap (>64 nm spacing between each nanorod peak), ensuring limited crosstalk and supporting robust multiplexing capabilities. Additionally, the absorption-to-scattering ratio across all geometries was notably high, with ∼90% of extinction attributed to absorption, underscoring their potential for strong PA contrast generation. Electric field simulations also revealed substantial electric field localization at nanorod tips (Figure S2), highlighting potential vulnerability to photothermal instability due to intense localized heating, which could cause structural deformation or melting during high-intensity illumination. These findings informed subsequent optimization of AuNR structure, photostability, and performance, aligning computational predictions with experimental design and validation.

### Experimental Synthesis and Morphological Characterization of AuNRs

Guided by computational insights, we synthesized a set of four distinct AuNR populations (AuNR1–AuNR4), each featuring precisely tunable longitudinal surface plasmon resonance (LSPR) peaks spanning approximately 650 to 950 nm. Transmission electron microscopy (TEM) characterization confirmed progressive increases in aspect ratio and morphological homogeneity within each batch (Figures 1C, S3). During synthesis, clear colorimetric transitions were observed, shifting progressively from grayish-blue for shorter rods (AuNR1), through purple (AuNR2), to paler red for longer rods (AuNR3 and AuNR4) (Figure 1D). These visually observable color changes correspond directly to increasing aspect ratios and associated plasmon resonance peaks. UV–Vis–NIR absorbance spectroscopy revealed well-defined, spectrally discrete LSPR peaks at approximately 665 nm (AuNR1), 730 nm (AuNR2), 820 nm (AuNR3), and 910 nm (AuNR4), in good agreement with computational predictions, and with at least 65 nm spacing between each nanorod peak (Figure 1E). Quantitative analysis from TEM images (>100 individual rods per sample) showed a systematic and precisely controlled increase in nanorod length from AuNR1 through AuNR4 (∼54 to 82 nm) (Figure 1F). Concurrently, rod width was inversely modulated, decreasing progressively (∼22 to 17 nm) to maintain nearly constant particle volume across all four AuNR subtypes (Figure 1G). The TEM measurements revealed aspect ratios increased progressively from approximately 2.4 (AuNR1) to about 4.8 (AuNR4) (Figure 1H). This precise morphological tuning was achieved through careful control of reaction parameters, primarily solution pH, silver ion concentration, and seed concentration, to generate elongated nanorods with progressively red-shifted resonances while avoiding excessively thin or fragile geometries. Extending the longitudinal plasmon resonance further into the near-infrared-II (NIR-II, ∼1000 to 1700 nm) region would require higher aspect ratios, which are increasingly difficult to synthesize uniformly while preserving narrow spectral features and structural robustness. Consequently, the resulting nanoparticles were intentionally optimized for robust and reproducible imaging within the NIR-I optical window.

### Silica Coating and PEGylation for Enhanced Photostability and Biocompatibility

Considering computational predictions regarding photostability vulnerabilities and existing literature highlighting stability concerns of bare AuNRs, we applied a protective mesoporous silica coating to enhance the thermal and structural robustness of the synthesized AuNRs.^35,44–46,68^ To control silica shell thickness, we designed and developed a hybrid precursor strategy using tetramethyl orthosilicate (TMOS) to initiate the reaction and tetraethyl orthosilicate (TEOS) to sustain growth (Figure S4). This TMOS-TEOS coprecursor approach allowed precise adjustment of hydrolysis and condensation rates, enabling fine control over shell thickness. Consequently, the final nanoparticles had uniform overall dimensions despite variations in the underlying AuNR core sizes. Subsequent PEGylation of these silica-coated AuNRs was carried out to reduce biofouling and further improve particle stability, biocompatibility, and circulation times in biological environments. TEM characterization verified uniform silica encapsulation around each AuNR subtype (Figure 1I). The resulting PEGylated silica-coated AuNRs (PS-AuNRs) displayed consistent dispersibility in aqueous solutions, visually confirmed by transparent, aggregation-free colloidal suspensions with distinguishable coloration ranging from deep purple (PS-AuNR1) to progressively paler reddish hues (PS-AuNR2–4) (Figure 1J). UV–Vis–NIR spectroscopic analysis revealed modest redshifts (∼20–40 nm) in LSPR peaks post silica coating, shifting all peaks slightly deeper into the NIR window, which suitably aligns with the excitation range of the imaging system used for PA imaging (optical parametric oscillator, OPO, Vevo LAZR) (Figure 1K). Importantly, the final PS-AuNR probes maintained spectral separations >64 nm, significantly minimizing cross-channel interference during multiplexing (Figure 1K). Silica shell thickness was systematically tuned to normalize the overall dimensions of all PS-AuNRs, yielding final particles with consistent size distributions (∼109.7 ± 6.2 nm length and ∼74.7 ± 5.8 nm width) for all PS-AuNRs (Figure 1L,M). Shell thickness progressively decreased with increasing rod length, from approximately 29 nm for PS-AuNR1 (shortest rods) down to approximately 21 nm for PS-AuNR4 (longest rods) (Figure 1N). This systematic adjustment ensured uniform overall size profiles and closely matched surface chemistries, achieved through silica coating and PEGylation, despite differences in underlying AuNR dimensions. Consistent with these matched dimensions, DLS measurements showed comparable effective hydrodynamic diameters, while zeta potential measurements were closely matched and mildly negative (Figure S5). To assess batch-to-batch reproducibility, PS-AuNR1–4 were synthesized in three independent batches and characterized by UV–Vis–NIR spectroscopy and TEM. Across batches, the longitudinal LSPR peak positions remained closely matched for each formulation, and TEM confirmed consistent nanorod morphology (Figure S6). This carefully engineered design isolates optical properties as the sole differentiating factor among the PS-AuNRs, making them ideally suited for multiplexed PA imaging. By controlling for these physicochemical factors, the only differentiating property among the PS-AuNRs is their optical spectrum, enabling accurate spectral unmixing and reliable multiplexed PA imaging in complex biological environments.

### PA Characterization of PS-AuNRs

To systematically evaluate PA imaging performance of the synthesized PS-AuNRs (PS-AuNR1 through PS-AuNR4), we employed a custom-designed, 3D-printed polyethylene tube phantom. Each PS-AuNR solution was adjusted to an identical optical density (OD) at its respective peak absorption wavelength: approximately 690 nm (PS-AuNR1), 770 nm (PS-AuNR2), 870 nm (PS-AuNR3), and 940 nm (PS-AuNR4) and added to different tubes for imaging. Initial qualitative PA imaging was then performed at these corresponding peak wavelengths. Images captured at these distinct wavelengths demonstrated clear differentiation among the four PS-AuNR subpopulations (Figure 2A, panels 1–4). At 690 nm, the PA image revealed an intense signal from PS-AuNR1, moderate intensity from PS-AuNR2, minimal signal from PS-AuNR3, and no detectable signal from PS-AuNR4 (Figure 2A, panel 1). Conversely, imaging at 940 nm exhibited the strongest PA intensity for PS-AuNR4, moderate intensity for PS-AuNR3, minimal for PS-AuNR2, and negligible signal for PS-AuNR1 (Figure 2A, panel 4). Intermediate wavelengths (770 and 870 nm) similarly yielded distinct, wavelength-specific PA intensity patterns that correlated precisely with each nanorod’s optical absorption maxima. We next evaluated spectral specificity and multiplexing feasibility of the PS-AuNR probes by quantifying wavelength-specific crosstalk (Figure 2B) and spectral overlap (Figure 2C). Because the crosstalk metric is evaluated with respect to a specific measurement channel and the spectral-overlap metric is calculated relative to a reference spectrum, both heat maps are directional. At key imaging wavelengths (690, 770, 870, and 940 nm), the crosstalk matrix revealed minimal interference between AuNR1 and AuNR2 despite their high spectral overlap (0.81), suggesting effective spectral separation in practice. However, a substantial crosstalk of approximately 83% was observed between AuNR3 and AuNR4, which also coincided with their pronounced spectral overlap (0.736). This significant spectral overlap and associated high crosstalk between PS-AuNR3 and PS-AuNR4 could limit multiplexing accuracy and thus warrants careful consideration in subsequent imaging experiments. However, spectroscopic PA imaging revealed distinct, clearly resolved peaks corresponding accurately to each PS-AuNR population, closely aligning with their respective UV–Vis–NIR absorbance profiles (Figure 2D). To verify the quantitative reliability of these nanorods, linearity tests were conducted across four different concentrations (OD = 0.125, 0.25, 0.50, and 1.0) for each PS-AuNR subtype (Figure S7). The resulting PA signals exhibited strong linear correlations with probe concentration (*R* ^2^ = 0.995, 0.978, 0.992, and 0.988 for PS-AuNR1–4, respectively), confirming predictable concentration-dependent PA responses across all four probes (Figure 2E). Such robust linearity is essential for accurate quantification and provides confidence in the capability of these nanoparticles for reliable molecular imaging applications in vivo.

**Figure 2:**
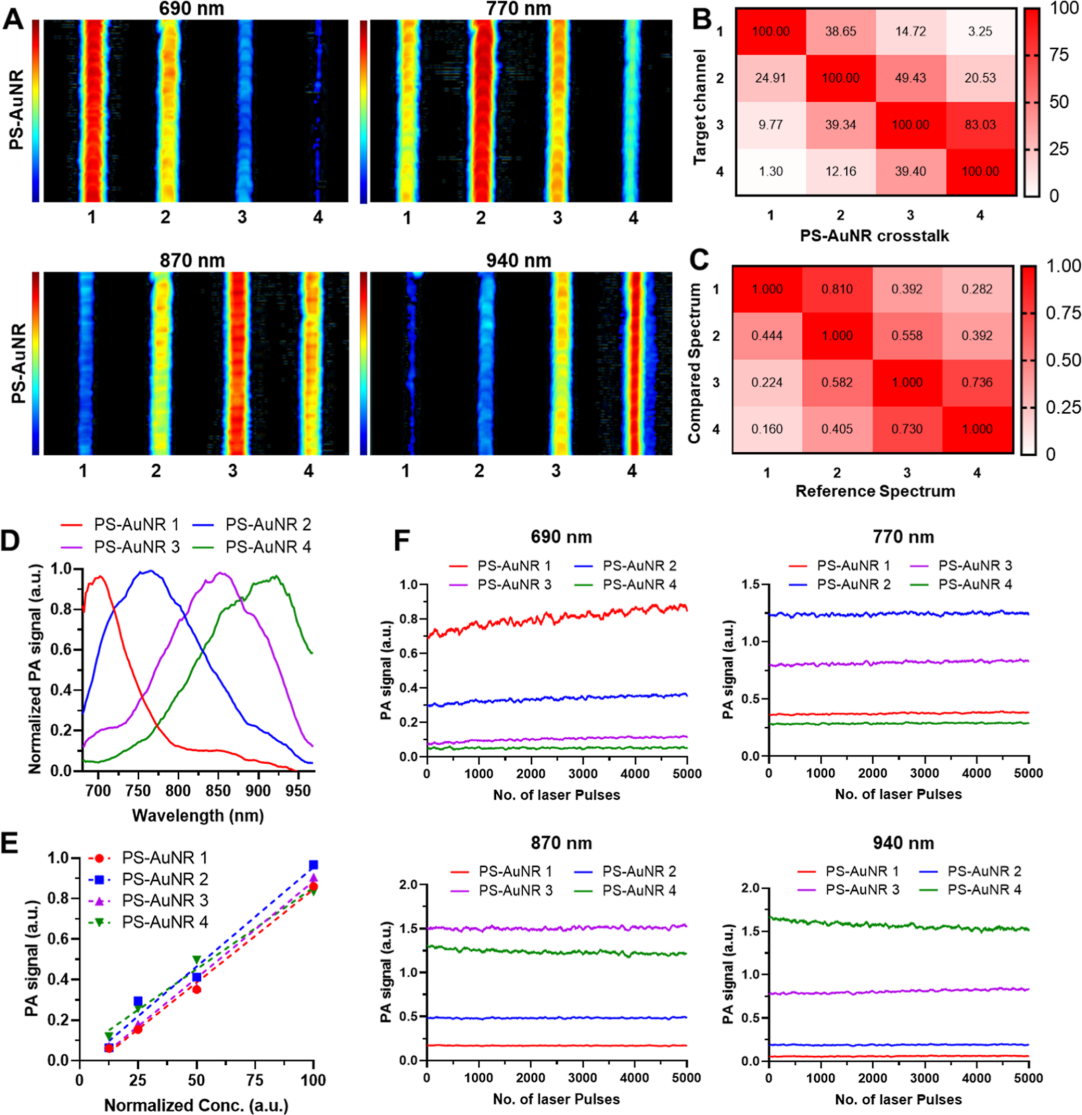
PA characterization of spectrally distinct AuNRs. (A). PA images of tube phantoms containing PS-AuNR1–4, acquired at four wavelengths (690, 770, 870, and 940 nm) approximately corresponding to the peak absorbance of each nanorod formulation. Each nanorod shows a differentiated PA signal at its respective wavelength, supporting potential for spectral multiplexing. (B). Directional crosstalk heat map between PS-AuNR1–4, illustrating the degree of PA signal overlap across the respective peak wavelengths. Each row is normalized to the intended target agent at that wavelength, such that the target channel is 100% and the remaining values indicate directional leakage from nontarget agents into that measurement channel. (C). Directional spectral overlap heat map quantifying pairwise similarity between normalized PA spectra of PS-AuNR1–4. Values close to 1 indicate high similarity relative to the reference PS-AuNR spectra. (D). Normalized PA spectra of PS-AuNR1–4 (λ = 680–970 nm), demonstrating spectrally separable profiles. (E). Linearity of PA signal as a function of optical density for each nanorod, measured at OD = 0.125, 0.25, 0.5, and 1.0, confirming strong linear correlation between concentration and PA amplitude (*R* ^2^ = 0.995, 0.978, 0.992, and 0.988 for PS-AuNR1–4, respectively). (F). Photostability of PS-AuNR1–4 at their respective peak wavelengths, assessed over 5000 laser pulses at ∼15 mJ/cm^2^ fluence. All formulations show stable PA signal output, indicating suitability for repeated and sustained imaging applications.

### Photostability of PS-AuNRs Under Pulsed Laser Excitation

We next evaluated the photostability of PS-AuNRs under prolonged pulsed laser excitation, an essential characteristic for reliable multiplexing and longitudinal imaging. Each PS-AuNR formulation was subjected to over 5000 laser pulses (∼15 mJ/cm^2^) at their respective peak absorption wavelengths (690, 770, 870, and 940 nm), with PA intensities monitored throughout the irradiation period. All four PS-AuNR populations exhibited excellent photostability, maintaining stable PA amplitudes with negligible signal loss or spectral shifts even after extended laser irradiation (Figure 2F). PA spectra measured before and after the photostability experiment for each PS-AuNR formulation remained closely matched, supporting minimal particle reshaping and strong potential for longitudinal imaging (Figure S8). This high photostability behavior is consistent with prior reports showing that mesoporous silica coating and the miniaturized rod dimensions mitigate laser and thermally induced structural deformation under extended PA imaging laser irradiation. In addition, it should be noted that laser fluence was not uniform across all tested wavelengths. Laser energy output was slightly lower (∼13 mJ/cm^2^) at the spectral extremes (700 and 940 nm) compared to the central wavelengths (∼18 mJ/cm^2^ at 770 and 870 nm). Collectively, these findings confirm that PS-AuNRs are robust against photothermal instability and maintain stable PA responses over lengthy imaging sessions or multiple imaging cycles.

### Quantitative Evaluation of PA Multiplex Imaging Accuracy

Building upon these characterizations, we next evaluated the spectral unmixing and multiplexing capabilities of our PS-AuNR platform by imaging a comprehensive set of nanoparticle mixtures (Figure 3A). In total, 31 independent mixtures comprising single-agent controls, binary, ternary, quaternary combinations, and various boundary conditions (Figure 3B; Table S1) were prepared and loaded into a polyethylene tube phantom. PA spectra were acquired across the full spectral range (680–970 nm, 2 nm increments), and spectral data were analyzed using a custom-developed spectral unmixing algorithm based on non-negative least-squares (NNLS) (Figures 3C; S9,S10). The algorithm quantitatively decomposed the acquired multiplexed spectra into linear combinations of known, single-agent PS-AuNR reference spectra, enabling the determination of relative nanoparticle contributions within each mixture (Table S2). Six representative mixtures were selected for detailed spectral reconstruction in the main manuscript: three binary mixtures with varying spectral separation (Mix 5, 6, and 7), two ternary mixtures differentiating between the first three and last three nanoparticles (Mix 11 and 14), and one quaternary mixture (Mix 15). Reconstructions of these mixture spectra using algorithm-derived nanoparticle contributions closely matched the experimentally obtained spectra across all selected mixtures (Figures 3D; S11,S12). Algorithm-generated estimations of nanoparticle contributions closely matched the experimentally defined ground-truth compositions across the mixtures, underscoring the reliability of our spectral unmixing method (Figures 3E; S13,S14). Heatmap visualization depicting differences between ground-truth and estimated compositions clearly illustrated algorithm performance across all mixtures (columns: PS-AuNR1–4; rows: Mixture 1–31) (Figure 3F). Low-difference regions indicated precise agreement, while higher-difference regions pinpointed spectral overlap challenges. A quantitative analysis of the mean absolute error (MAE %) per mixture further clarified these performance trends (Figure 3G). As expected, binary mixtures containing spectrally distant nanoparticles (e.g., Mix 7: PS-AuNR1 and 4) exhibited exceptionally low MAE rates (<0.5%), confirming the robustness and precision of the approach for straightforward multiplex scenarios. Generally, we see that the spectral overlap determines multiplexing accuracy. As expected, mixtures featuring nanoparticles with lower overlap, such as mixture 5 (PS-AuNR1 and 2) have lower errors (<3.5%), while mixtures with greater spectral overlap, such as mixture 10 (PS-AuNR3 and 4), yield higher estimation errors (∼11.5%), indicative of increased signal crosstalk (Figure 3G). Across all mixtures and nanoparticles, the average MAE was 4.30%, a highly acceptable error margin for multiplexed molecular imaging. Next, evaluated the performance of each individual contrast agent across all the mixtures (Figure 3H). AuNR3 exhibited the highest error, primarily due to its significant spectral overlap with AuNR4 (5.42%), whereas AuNR2 demonstrated the lowest error due to its clear spectral separation from both AuNR1 and AuNR3 (3.07%). When analyzing MAE as a function of mixture complexity (binary, ternary, or quaternary), interestingly, we observed no substantial increase in error as the number of simultaneously unmixed agents increased from two to four (Figure 3I). This suggests that multiplexing performance is not strictly limited by the number of agents, but rather by the specific spectral properties of the chosen agents. Supporting this, we observed wide variation in error across binary mixtures, highlighting the critical role of contrast agent selection (Figure 3I). To further explore this relationship, we examined how minimum spectral spacing between agents influences unmixing accuracy (Figure 3J). A clear inverse trend was observed: greater spectral separation between agents corresponded to lower MAE. This finding reinforces the importance of designing spectrally distinct probes to maximize multiplexing accuracy. Finally, we assessed whether contrast agent concentration within mixtures influenced multiplexing accuracy (Figure 3K). No clear relationship emerged between concentration and absolute error, demonstrating that multiplexing accuracy remains robust even under boundary conditions and highly skewed conditions. Collectively, these data establish that the PS-AuNRs, combined with our spectral unmixing approach, provide robust and quantitative multiplexed PA imaging suitable for complex molecular imaging applications.

**Figure 3:**
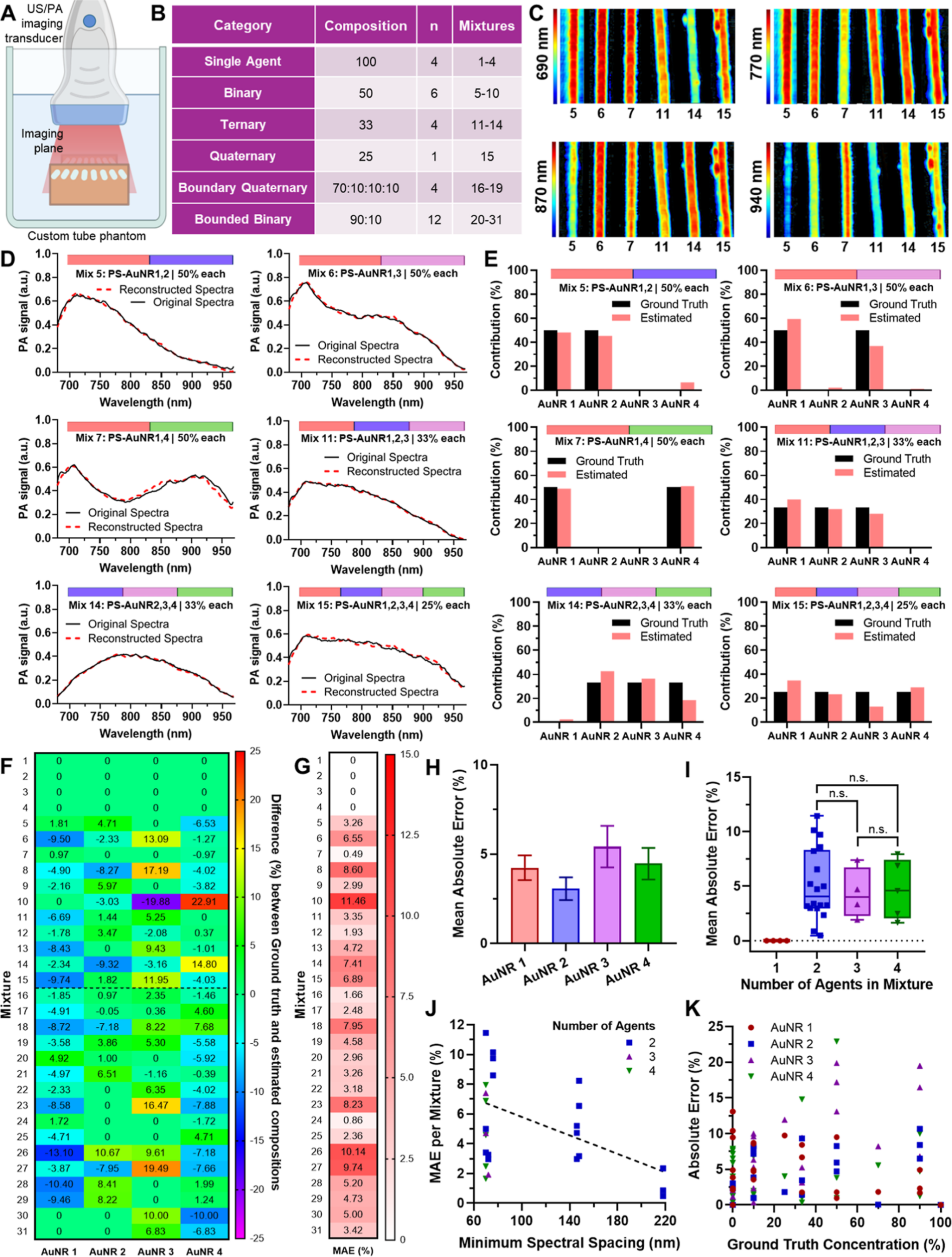
In vitro PA multiplexing of PS-AuNR mixtures. (A). Schematic of the PA imaging setup, illustrating the orientation of the tube phantom and the imaging plane. (B). Experimental design table showing 31 total nanorod mixtures spanning a full factorial comprehensive design space to evaluate multiplexing performance. The mixtures are categorized into six groups: single-agent, binary, ternary, quaternary, and two boundary conditions (a skewed binary at 90:10 and a skewed quaternary at 70:10:10:10). (C). Representative PA images of six selected mixtures: Mix 5 (AuNR1 + 2), Mix 6 (AuNR1 + 3), Mix 7 (AuNR1 + 4), Mix 11 (AuNR1 + 2 + 3), Mix 14 (AuNR2 + 3 + 4), and Mix 15 (AuNR1 + 2 + 3 + 4), captured at four wavelengths (690, 770, 870, and 940 nm). Distinct spectral separation and spatial distribution are visualized across mixtures with increasing complexity. (D). Comparison of original versus reconstructed spectra for the selected mixtures. (E). Quantitative comparison of ground truth and estimated contribution percentages for AuNR1–4 in the six selected mixtures. (F). Heat map of error values for each contrast agent across all 31 mixtures, reflecting the deviation between estimated and ground truth contributions. (G). Heat map of mean absolute error (MAE, in %) per mixture, capturing the overall unmixing performance across the experimental design space. (H). MAE % per contrast agent aggregated across all 31 mixtures, revealing agent-specific trends in performance. AuNR 3 shows higher error due to a large spectral overlap with AuNR4, whereas agents with greater spectral isolation exhibit lower error. (I). MAE % plotted against the number of agents in each mixture, showing no consistent trend in error with increasing spectral complexity, suggesting that multiplexing accuracy is independent of the number of agents in the mixture. (J). MAE % per mixture as a function of minimum spectral spacing between agents, demonstrating that increased spectral separation correlates with reduced error and improved unmixing accuracy. (K). Absolute error (%) for each contrast agent as a function of its ground truth contribution in each mixture. No consistent correlation was observed between agent concentration and error, indicating that detection accuracy is not solely dependent on abundance.

To assess the capabilities of PS-AuNRs in biologically relevant environments, we evaluated both their spectral distinguishability in the presence of strong endogenous optical background and their cytocompatibility under relevant exposure conditions. Cytotoxicity evaluation in mesenchymal stem cells showed that PS-AuNR1–3 did not significantly reduce viability at concentrations up to 16 OD, whereas PS-AuNR4 produced only a slight decrease at 16 OD; notably, PS-AuNR1–4 show no toxicity up to 8 OD, exceeding the concentrations used in this study (Figure S15). Next, each of the PS-AuNRs was combined with whole human blood and loaded into the tube phantom setup. UV–Vis–NIR spectroscopy (400–1100 nm) revealed that blood absorption dominated the 400–600 nm range, effectively obscuring signals from exogenous contrast agents in this region (Figure S16A,B). However, beyond approximately 600 nm, blood absorption sharply diminished, providing a clear spectral window. Within this NIR-I optical window, PS-AuNR absorption peaks remained clearly distinguishable amidst the blood background, confirming the practical utility of our spectral design (Figure S16C,D). Spectroscopic PA imaging of the PS-AuNRs in the presence of blood was conducted at 532 nm (Figure S17A,B) and across the same OPO wavelength range (680–970 nm) (Figure S17C–H). At 532 nm, blood generates a strong PA signal due to high hemoglobin absorption, significantly reducing the signal-to-background ratio and complicating contrast agent detection (Figure S17A,B). In contrast, within the OPO tuning range used for multiplexed imaging (690, 770, 870, and 940 nm), the intrinsic PA signal from blood is minimal relative to that of the PS-AuNRs (Figure S17C–H). However, this analysis does not account for other endogenous absorbers such as lipids or melanin, which may contribute to background signal in more complex biological tissues.

### In Vivo Subcutaneous Multiplexed PA Imaging of PS-AuNRs

Encouraged by the promising in vitro results, we next evaluated the multiplexing capability of PS-AuNRs in vivo. We utilized a hypodermal murine model in which each of the four distinct PS-AuNR subpopulations was individually suspended in Matrigel and injected subcutaneously into discrete locations on the dorsal region of mice (Figure 4A,B). We prepared a panel of 12 independent mixtures comprising 4 single-agent, 3 binary, 2 ternary, 1 quaternary, and 2 boundary-condition mixtures—one binary and one quaternary with skewed compositions (Figure 4C, Table S3). Each mixture was uniformly dispersed in Matrigel and injected subcutaneously. Five independent biological replicates were performed per mixture group, resulting in a comprehensive in vivo data set for evaluating multiplexing performance (60 total groups). Spectroscopic PA imaging (680–970 nm, 2 nm steps) and 3D PA imaging were performed. US/PA images revealed distinguishable PA signal profiles across all injected PS-AuNR formulations, enabling clear spatial discrimination between mixtures. Representative images from each of the 12 mixture groups were organized across three sets: single-agent mixtures (mixtures 1–4, Figure 4D), binary and quaternary mixtures (mixtures 5–8, Figure 4F), and ternary and boundary-condition mixtures (mixtures 9–12, Figure 4H). Each panel contains four subimages acquired at the corresponding wavelengths close to the peak (680, 750, 860, and 960 nm). Due to mild spectral distortion in tissue, we prioritized wider interchannel spacing to reduce crosstalk even if that was not the absolute PS-AuNR peak. As expected, PA signal intensity varied across wavelengths, with strong responses from the matched nanoparticle at its corresponding peak (Figure 4D, F, H). Consistent with the favorable NIR-I imaging window, raw PA spectra extracted from depth-matched tissue background ROIs remained low across the full acquisition range relative to the PS-AuNR boluses, typically by approximately an order of magnitude at the nanoparticle signal maxima (Figure S18). Notably, in the quaternary mixture, all four nanoparticles produced detectable signals at their respective wavelengths, while binary mixtures showed enhanced contrast at the relevant two wavelengths (Figure 4F). To validate spatial unmixing performance, we applied NNLS-based spectral decomposition to generate agent-specific maps. Dominant agent maps, indicating the nanoparticle with the highest contribution per pixel, are shown for all four agents across the same injection sets (Figure 4E, G, I). For visualization, each PS-AuNR was assigned a distinct color: PS-AuNR1 (red), PS-AuNR2 (blue), PS-AuNR3 (purple), and PS-AuNR4 (green). These maps demonstrate strong qualitative agreement with the expected composition of each mixture. Single-agent injections yield highly specific spatial localization, while binary, ternary, and quaternary mixtures show colocalized signals consistent with the injected combinations (Figure 4E, G, I). In addition to single-agent maps, we generated two merged representations to visualize spatial composition across all injection sites: a dominant-agent composite map and an additive color integration map. The dominant-agent map assigns each pixel to its most abundant nanoparticle, while the additive map layers spectral contributions with weighted color intensities (Figure 5). Together, these representations clearly distinguish the spatial distribution of PS-AuNRs within each injection bolus, revealing the expected composition in all 12 mixture groups. As controls, saline-only and Matrigel-only injections were included to assess background interference. Neither condition produced detectable signal in raw PA images, unmixed spectral maps, or merged reconstructions, confirming that endogenous PA signal or the matrix components did not contribute to unmixing results (Figure S19A–D).

**Figure 4:**
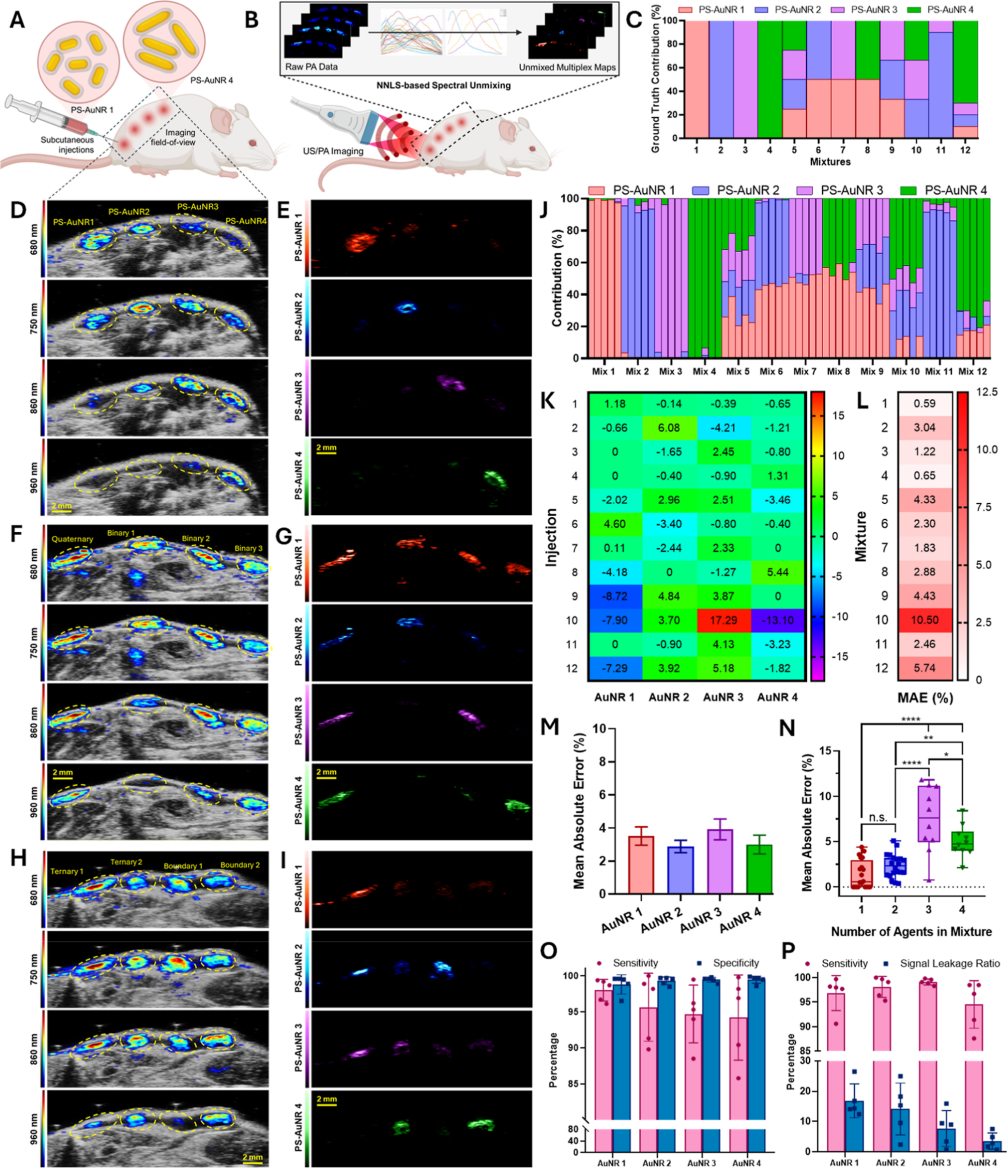
In vivo PA multiplexing of PS-AuNR mixtures in a subcutaneous injection murine model. (A). Schematic of the subcutaneous injection model showing four injection sites in the dorsal region of each mouse. (B). Schematic of the US/PA imaging setup and spectral unmixing strategy using NNLS to generate spatially resolved contrast agent maps. (C). Experimental design depicting 12 distinct injection groups: four single-agent controls, one quaternary mixture, three binary mixtures, two ternary mixtures, and two boundary conditions (a skewed binary and a skewed quaternary). Ground truth nanorod contributions for each mixture are shown as a bar graph. Each group includes *n* = 5 mice (biological replicates). (D). Representative US/PA images of single-agent injections at 680, 750, 860, and 960 nm, selected to approximate the peak absorbance of each nanorod formulation. (E). Multiplexed spatial maps of individual nanorod contributions for the same single-agent injections shown in Panel D. Each image corresponds to one of AuNR1–4 (arranged top to bottom with respective colormaps) and demonstrates clear localization of each nanorod at its designated injection site, with minimal signal from the other nanorods in those regions. (F). Representative US/PA images of four additional experimental groups: one quaternary mixture and three binary combinations. (G). Corresponding multiplexed maps of AuNR1–4 for the groups shown in Panel F. All four nanorods are detected in the quaternary injection site, while only the respective two nanorods are visible in each of the three binary injection sites. (H). US/PA images of ternary mixture groups and two boundary condition groups. (I). Multiplexed contribution maps of AuNR1–4 for the groups shown in Panel H, showing spatial localization of the expected nanorods at the respective injection sites for both ternary and boundary mixtures. (Scale bar, 2 mm. Applies to all image panels). (J). Stacked bar graph showing estimated contribution percentages of AuNR1–4 across all 60 replicates (12 experimental groups, *n* = 5 each). Each bar represents the multiplexed output for a single replicate, demonstrating strong agreement with the ground truth design in Panel C. (K). Heat map of average error values for AuNR1–4 across all 12 mixtures (averaged over *n* = 5 mice). Most mixtures show low error <5%, with the highest error observed in Mix 10 (ternary), which includes both AuNR3 and AuNR4, nanorods with maximum spectral overlap. (L). Mean absolute error percentage per mixture. Errors range from 0.60% to a maximum of 10.5%, with the majority of mixtures demonstrating accurate unmixing. (M). Mean absolute error percentage per contrast agent aggregated across all mixtures (*n* = 5 each). AuNR3 shows the highest error due to spectral proximity with AuNR4, while other agents exhibit lower error rates. (N). Mean absolute error percentage grouped by the number of agents per mixture. While ternary mixtures display the highest overall error, no consistent trend is observed across mixture complexity. Quaternary mixtures, despite containing four agents, exhibit relatively low error, highlighting the influence of spectral spacing over agent count. (O). Spatial accuracy of multiplexed imaging quantified via per-agent sensitivity and specificity based on dominant-agent map predictions relative to known injection sites. All PS-AuNRs exhibited high sensitivity (>94%) and specificity (>98%) across five replicates per condition, indicating robust localization with minimal off-target signal. (P). Intensity-based accuracy metrics showing per-agent sensitivity and signal leakage ratio. PS-AuNR1 and PS-AuNR2 showed higher leakage, likely due to endogenous background at lower wavelengths, while PS-AuNR3 and PS-AuNR4 showed minimal leakage. Intensity sensitivity remained high (>94%) across all agents.

**Figure 5:**
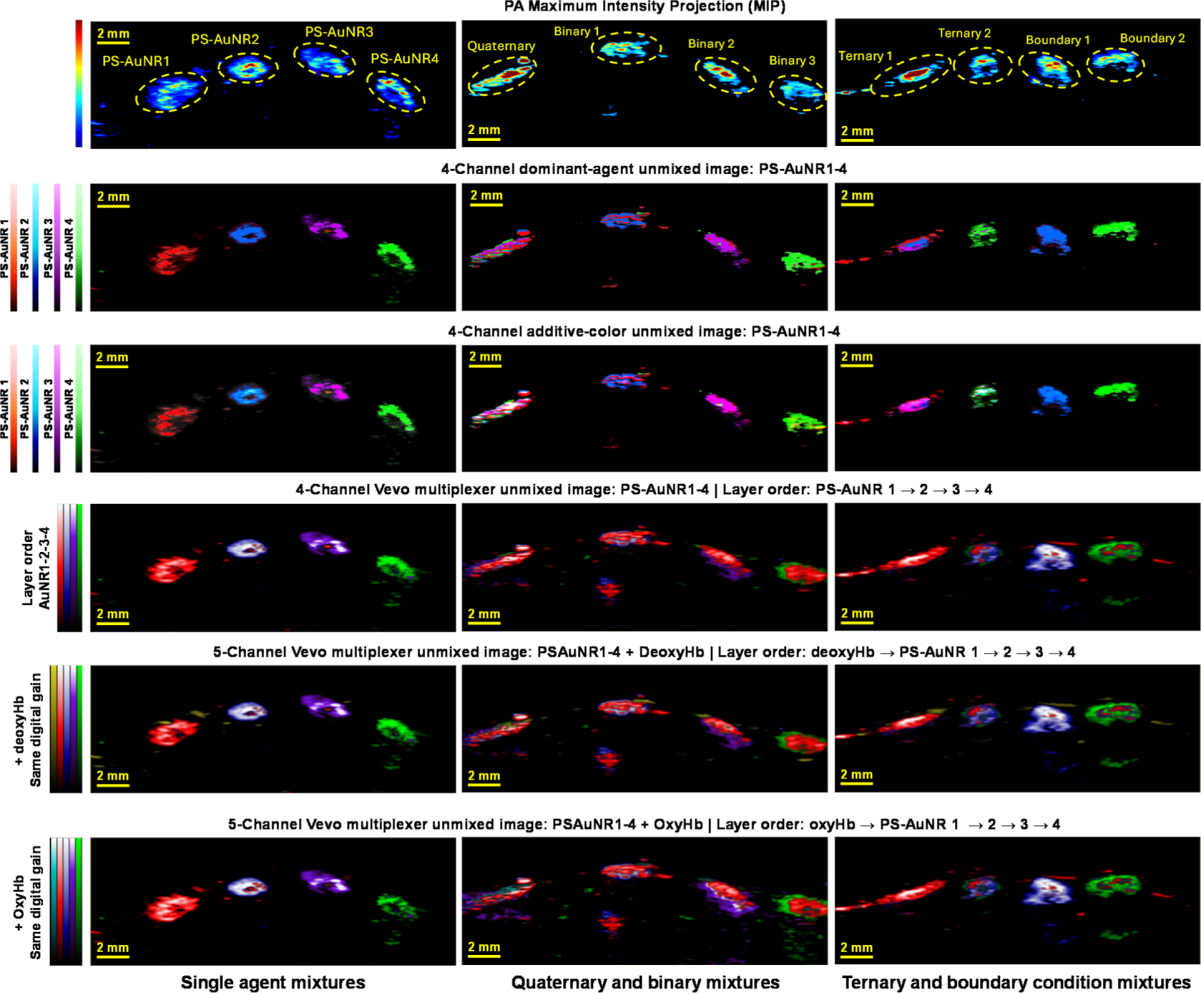
PS-AuNR multiplexing in the presence of endogenous absorbers using the native VevoLAB unmixing algorithm. Representative in vivo subcutaneous spectroscopic PA data sets: Column 1, single-agent mixtures; Column 2, quaternary and binary mixtures; and Column 3, ternary and boundary-condition mixtures. (A) PA MIP images and the composition of each injection bolus. (B) Four-channel dominant-agent maps generated using the custom NNLS-based unmixing workflow. (C) Four-channel additive-color NNLS maps, in which pixel colors reflect the combined relative contributions of multiple agents. (D) Four-channel unmixed images generated using the built-in VevoLAB multiplexer workflow with the fixed layer order PS-AuNR1–2–3–4. (E) Five-channel VevoLAB unmixed images generated with deoxyhemoglobin added as an endogenous absorber channel and displayed with the layer order deoxyhemoglobin followed by PS-AuNR1–2–3–4. (F) Five-channel VevoLAB unmixed images generated with oxyhemoglobin added as an endogenous absorber channel and displayed with the layer order oxyhemoglobin followed by PS-AuNR1–2–3–4. Across all panels, PS-AuNR1, PS-AuNR2, PS-AuNR3, and PS-AuNR4 are shown in red, blue, purple, and green, respectively, while deoxyhemoglobin and oxyhemoglobin are shown in yellow and teal. This comparison demonstrates that the PS-AuNR platform remains visually interpretable within a standard commercial unmixing workflow and retains clear multiplexed contrast even when endogenous absorber channels are included.

Quantitative spectral unmixing was performed within defined regions of interest (ROIs) for each injection using our NNLS-based algorithm, yielding estimated agent-wise contributions for every replicate. Estimated contributions of the four PS-AuNR agents were directly compared to the known ground truth compositions across all subcutaneous mixtures. Figure 4J presents a stacked bar plot of the estimated compositions for all 60 subcutaneous injections (12 mixtures × 5 replicates), visually recapitulating the ground-truth pattern shown in Figure 4C (Figure 4J, C). This close correspondence highlights the robustness of our unmixing algorithm in accurately calculating complex mixture compositions in tissue (Figure S20). Reconstructions of the spectral signatures using algorithm-derived nanoparticle contributions closely matched the experimentally obtained spectra across all experimental conditions (Figure S21). A heatmap illustrating errors (mean; *n* = 5 replicates) across each injection further highlighted the robustness of the multiplexing strategy (Figure 4K). A MAE % summary heatmap demonstrates error magnitudes per mixture, with most remaining below 5%, except Mixture 10 (comprising AuNR2, AuNR3, and AuNR4) exhibiting high errors of ∼10.5%, likely due to spectral overlap between AuNR3 and AuNR4 (Figure 4L). Analysis across all subcutaneous injections yielded a MAE % of ∼3.3%, indicative of excellent accuracy in spectral unmixing within in vivo scenarios (Table S4). Next, we examined agent-wise error trends across all injections. As in the in vitro studies, AuNR3 exhibited slightly elevated error due to spectral interference from AuNR4, while AuNR2 maintained the lowest MAE (Figure 4M). To evaluate the effect of mixture complexity, we analyzed MAE as a function of the number of agents (single, binary, ternary, and quaternary). Contrary to the in vitro data, mixtures containing three or four agents displayed higher error than binary cases (Figure 4N). Interestingly, four-agent mixtures exhibited slightly lower MAE than ternaries, suggesting that, similar to the in vitro case, spectral spacing and agent selection, rather than simply the number of components, are the dominant factors influencing unmixing accuracy (Figure 4N). Consistent with this, a correlation analysis between minimum spectral spacing and MAE confirmed an inverse relationship (Figure S22A). Conversely, no clear association was observed between MAE and the relative contribution of each agent, indicating that unmixing accuracy remains robust even under boundary or skewed compositions (Figure S22B).

To evaluate the fidelity of our multiplexed imaging approach, we independently assessed spatial and intensity-based accuracy for each PS-AuNR. To evaluate the localization of identified contrast agents, spatial accuracy was quantified. Sensitivity and specificity per agent were computed on a pixel-wise basis based on dominant-agent map predictions relative to known injection ROIs (Figure 4O). For each agent, sensitivity captured the fraction of ground-truth pixels correctly classified, while specificity measured the proportion of background pixels correctly identified as negative. All four PS-AuNRs exhibited high sensitivity (>94%) and specificity (>98%), indicating robust spatial localization and minimal false-positive assignment (Figure 4O). Complementing this, we quantified intensity-based accuracy which incorporated the actual unmixed PA signal values (rather than binary pixel presence) into performance quantification. Intensity-based sensitivity was calculated as the fraction of total PA signal correctly recovered within the true region. To account for off-target signal leakage, we report the Signal Leakage Ratio (SLR), defined as the proportion of erroneously assigned signal found outside the annotated ground truth ROI (Figure 4P). While intensity-based sensitivity remained high (>94%), notably, AuNR1 and AuNR2 exhibited higher signal leakage ratios (∼16.8% and ∼14.1% respectively), suggesting a greater degree of signal misclassification in the lower wavelength regions where interference from endogenous tissue PA signal is more prominent. Together, these spatial and intensity analyses provide a complementary framework to benchmark unmixing performance in terms of both localization and quantitative recovery of PA signal. These results collectively validate the utility of our spectrally tunable PS-AuNR platform for quad-channel in vivo multiplex PA imaging, highlighting their potential to accurately detect and monitor multiple biomarkers simultaneously in biologically relevant settings.

### Comparison with Commercial VevoLAB Unmixing and Hemoglobin-Channel Analysis In Vivo

To further assess practical usability and endogenous-background robustness, we compared our custom NNLS-based workflow with the built-in VevoLAB spectral deconvolution workflow using representative in vivo subcutaneous data sets (Figure 5A). In the single-agent, quaternary/binary, and ternary/boundary-condition examples, the dominant-agent and additive-color NNLS maps recovered the expected PS-AuNR compositions, with clear localization of the corresponding boluses (Figure 5B,C). The commercial VevoLAB workflow likewise recovered the major multiplexed signal patterns (Figure 5D), although its layered rendering format made visual prominence partly dependent on display order. This was most apparent in mixed boluses containing PS-AuNR1, where the fixed display order caused the PS-AuNR1 layer to visually dominate regions in which other channels were also present. At the same time, the layered rendering preserved low-level boundary signal and produced an intuitively useful practical visualization of the multiplexed data (Figure S23A,B).

We then added deoxyhemoglobin and oxyhemoglobin as additional channels within the VevoLAB workflow to directly assess endogenous interference under realistic in vivo conditions (Figure 5E,F). Under matched visualization settings, both endogenous hemoglobin channels remained weak relative to the PS-AuNR boluses and did not materially obscure nanoparticle localization. When the hemoglobin channels were displayed with substantially increased brightness, low-level endogenous signal became more apparent, but it remained broadly distributed and spatially distinct from the localized nanoparticle contrast (Figure S24A–C). Together, these results show that the PS-AuNR library can be resolved not only with a simple custom NNLS workflow but also within a standard commercial software environment while remaining robust to endogenous oxyhemoglobin and deoxyhemoglobin background across the NIR-I multiplexing range.

## Discussion

This work doubles PA multiplexing from the previously limited two channels to four channels, utilizing a rationally engineered library of PS-AuNRs coupled with minimal setup requirements comprising a single commercial OPO laser and a simple NNLS unmixing algorithm. The AuNRs have final LSPR peaks within the 680–970 nm range, which corresponds to the full tuning range of a single OPO laser. Additionally, controlled silica-coating and PEGylation of the AuNRs resulted in similar sized probes with analogous surface chemistries, ensuring comparable kinetics and biodistribution. A basic NNLS algorithm is sufficient to unmix the four channels because each probe’s spectrum is both narrow and well separated. Compared to existing PA multiplexing approaches that utilize multiple lasers setups, temporal acquisition complexity, multimodal imaging, and complicated unmixing algorithmic pipelines, we double the current multiplex capabilities without imposing hardware or computational complexity. Despite this streamlined workflow, the platform achieves an average MAE of ∼4.3% in vitro and of ∼3.3% in vivo while multiplexing four contrast agents simultaneously.

More broadly, the key contribution of this work is platform-level rather than material-level. AuNRs themselves are already well established as PA contrast agents, and prior studies have demonstrated the utility of silica-coated AuNRs for PA imaging and proof-of-concept multiplexing. However, most previous reports have focused on single-agent imaging, two-agent discrimination, or narrower demonstrations without explicit benchmarking against known mixture compositions. In contrast, our study integrates four spectrally engineered AuNR probes with matched silica-PEG surface chemistry, a standard commercial OPO-based imaging system, and a simple linear unmixing workflow, while quantitatively validating performance against controlled ground-truth mixtures in both phantom and in vivo experiments. This combination of expanded channel count, physicochemical matching across probes, hardware accessibility, and explicit quantitative benchmarking is, to our knowledge, not established in prior PA multiplexing studies and is important for enabling broader adoption in application-driven biological imaging. The added oxyhemoglobin and deoxyhemoglobin analyses further suggest that the spectral framework can remain interpretable even as additional endogenous channels are introduced, providing a practical step toward larger panel multichannel PA imaging.

Despite these advantages, the approach has important limitations and questions that are still unanswered. Spectral congestion between PS-AuNR3 and PS-AuNR4 produced very high multiplexing errors, indicating that further peak narrowing by improving AuNR homogeneity or wider spectral spacing between probes may be necessary for applications requiring tighter error tolerances. Our spectral unmixing framework also assumes linear mixing and uniform optical fluence; it does not account for depth-dependent attenuation, wavelength-dependent fluence variations, aggregation-induced plasmon coupling, or other nonlinear photothermal effects, all of which may introduce additional error in highly scattering or deeply embedded tissues. Although silica coating and PEGylation improve colloidal stability, aggregation induced plasmon coupling in biological environments could broaden spectra and should be monitored for longitudinal imaging applications. Moreover, in vivo validation was restricted to subcutaneous implants, and long-term biodistribution or clearance of the probes was not assessed. Despite the favorable biocompatibility profile of gold nanomaterials in short-term studies, their long-term fate remains a key translational hurdle, therefore, comprehensive pharmacokinetic and toxicology studies extending to several months are essential before clinical deployment.

Future work will pursue both improvement of the multiplexing platform and application toward clinically relevant questions. First, by exploiting lasers with broader tuning ranges, such as 600 to 1100 nm and beyond, additional AuNR probes could be incorporated into the multiplexing framework, enabling larger multiplex panels of six to seven simultaneous channels. Broader excitation range could be utilized with the present four-channel multiplexing, wherein we extend the peak separations of PS-AuNRs from ∼65 nm to >100 nm further reducing crosstalk and estimation error. Extension into the NIR-II region may be particularly valuable in this regard, given its favorable tissue-optical properties. However, for AuNR-based probes, shifting plasmon resonances further into NIR-II generally requires higher aspect-ratio nanorods, which can be more difficult to synthesize reproducibly while maintaining narrow absorption bands, structural robustness, and photostability. Second, while we intentionally used NNLS as a simple and transparent baseline unmixing method, more advanced frameworks such as non-negative matrix factorization or machine learning-based approaches may further improve performance in settings with stronger fluence variation, more complex backgrounds, or larger multiplexing panels.

Now, in terms of utilization of our multiplexing platform, the uniform silica surface provides a convenient scaffold for attaching antibodies, peptides, or small molecule ligands. This enables multi biomarker mapping of tumor heterogeneity, real time monitoring of cellular dynamics in adoptive cell therapy, and other complex biological events in three dimensions. A compelling future application of four-channel PA multiplexing is the spatial classification of heterogeneous breast cancers through concurrent imaging of ER, PR, HER2, and EGFR.^69^ This panel spans the principal clinical determinants of breast cancer subtype while incorporating a complementary marker associated with basal-like disease, raising the possibility of resolving mixed phenotypes and spatially distinct receptor-defined compartments within a single tumor.^69,70^ Such an approach could be particularly valuable where intratumoral heterogeneity or primary-to-metastatic receptor discordance limits the representativeness of conventional biopsy-based classification.^70–73^ More broadly, this type of multiplexed PA imaging could reveal how distinct cellular subtypes are spatially organized within tumors, including preferential localization to the core or periphery. In cell therapy contexts, simultaneous visualization of multiple cell types could provide mechanistic insight into synergy, competition, or dominance among therapeutic populations.

Together, this platform represents a step forward in making PA multiplexing more scalable, accessible, and biologically informative. By balancing spectral precision with practical simplicity, this platform lays the groundwork for future applications where high-fidelity, multitarget molecular imaging at depth is essential.

## Conclusions

In this study, we developed and validated a platform for four-channel PA multiplexing using a library of chemically and geometrically identical yet spectrally distinct PS-AuNRs coupled with minimalistic hardware and computational infrastructure. Through a reproducible three-step seed-mediated synthesis, we achieved precise control over nanorod dimensions, yielding particles with narrow, discrete plasmon resonances across the NIR-I optical window. Hybrid silica-coating and PEGylation produced contrast agents with enhanced photostability, minimal aggregation, and uniform morphologies, without compromising spectral separability. The resulting PS-AuNRs demonstrated robust multiplexing capabilities, enabling accurate spectral unmixing with low mean absolute errors across a wide range of in vitro (<4.3%) and in vivo (3.3%) conditions. Importantly, the platform remained practically interpretable within both a simple custom NNLS workflow and the built-in commercial VevoLAB unmixing software, while showing minimal interference from added oxyhemoglobin and deoxyhemoglobin channels. While the platform significantly reduces hardware and algorithmic complexity compared to existing PA multiplexing strategies, challenges remain. These include improving spectral separability between closely spaced probes, addressing depth-dependent optical attenuation and nonlinear thermal effects, and evaluating long-term biodistribution and biocompatibility. Future work will focus on integrating advanced unmixing frameworks, expanding the spectral palette using broader laser tuning ranges, and functionalizing the probes for molecular targeting. By enabling simultaneous detection of multiple molecular species with high fidelity and minimal system complexity, this work lays the foundation for scalable, real-time PA multiplexing. The platform holds strong potential for advancing in vivo molecular diagnostics, including high-resolution mapping of tumor heterogeneity and monitoring multicellular dynamics in therapeutic applications.

## Methods and Experimental

### Materials

Hexadecyltrimethylammonium chloride (CTAC, 25% w/w aqueous solution), 1-decanol (*n*-decanol, 98%), hydrogen tetrachloroaurate trihydrate (HAuCl_4_·3H_2_O, ≥99.9%), silver nitrate (AgNO_3_, ≥99.0%), hydroquinone (≥99.0%), l-ascorbic acid (≥99%), hydrochloric acid (HCl), nitric acid (HNO_3_), tetraethyl orthosilicate (TEOS), tetramethyl orthosilicate (TMOS), sodium hydroxide (NaOH), methanol, and sodium borohydride (NaBH_4_, 99%) were purchased from Sigma-Aldrich and used without any further modifications. Cetyltrimethylammonium bromide (CTAB, VWR), m-PEG-silane (MW: 5000, Creative PEGWorks). Milli-Q grade water (resistivity 18.2 MΩ cm at 25 °C) was used in all experiments.

### Ultrasound and Photoacoustic Imaging

All experiments were performed on a commercial Vevo LAZR system equipped with a flashlamp-pumped Q-switched Nd/YAG laser with second-harmonic generation and an optical parametric oscillator (OPO), operating over 680–970 nm with 2 nm step size, 20 Hz repetition rate, 7 ns pulse duration, 45 ± 5 mJ peak pulse energy at 20 Hz, and an illumination spot size of 24 mm2 (1 mm × 24 mm). We also include the relevant system-level PA specifications reported by the manufacturer, including ∼70 dB dynamic range, PA SNR of ∼20 ± 10 dB, and acquisition times of approximately 0.2 s for 2D and 74 s for a 10 mm 3D scan.

### Ethics Approval

Ethics approval for experiments reported in the submitted manuscript on animal or human subjects was granted. Human discard blood was obtained by the Wilbur Lam Lab in clinical studies approved by the Georgia Institute of Technology Institutional Review Board (IRB No. H23498). Informed consent to collect blood was obtained by all participants to participate in the study. All animal procedures were approved by the Institutional Animal Care and Use Committee (IACUC) at the Georgia Institute of Technology under protocol A100284 in accordance with federal guidelines for the care and use of laboratory animals.

### Finite-Difference Time-Domain Simulations of AuNRs

Optical properties of four distinct AuNRs were calculated via a FDTD simulation (Lumerical Inc.). The dielectric constant and reflective index of gold were taken from the value in Johnson and Christy, while surrounding medium was set as a water and surrounding temperature was set as 300 K. A total-field/scattered-field source with a spectral pulse from 600 nm to 1000 nm was employed to simulate the light-AuNR interaction. To obtain optical cross sections for each AuNR, the average values considering light illumination at different light polarization angles, including p-polarization and s-polarization were calculated. The electric field distribution from each AuNR was obtained along its longitudinal axis to highlight the localization of the electric field at the tip regions. These results informed the optimization of their design for PA imaging applications.

### Synthesis of Miniature Tunable AuNRs

A three-step synthesis procedure was used to synthesize the miniature tunable AuNRs according to a previously published method with some modifications.^65^ Two growth solutions were prepared by dissolving CTAB (50 mM, 9.111 g) and *n*-decanol (13.5 mM, 1.068 g for the AuNR seed OR 11 mM, 870.5 mg for the tunable AuNRs) in 500 mL of Milli-Q grade water. The solution was sonicated at ∼60 °C for 1 h to ensure the complete incorporation of *n*-decanol into CTAB micelles. The resulting solution remained stable above 16 °C.

For the 1–2 nm Au-Seeds, HAuCl_4_ (0.05 M, 200 μL) and ascorbic acid (0.1 M, 100 μL) were added to the growth solution containing CTAB (50 mM, 20 mL) and *n*-decanol (13.5 mM), under gentle stirring at 25–27 °C. After 1–2 min, NaBH_4_ (0.02 M, 800 μL, freshly prepared) was injected under vigorous stirring. The solution transitioned from colorless to brownish-yellow, indicating seed formation. The mixture was aged for 60 min at 25–27 °C to ensure complete consumption of excess borohydride.

Then, for the synthesis of the small anisotropic Au-Seeds (21 × 7.5 nm), HCl (1 M, 21 mL), HAuCl_4_ (0.05 M, 3000 μL), and ascorbic acid (0.1 M, 3900 μL) were sequentially added to a solution containing CTAB (50 mM, 300 mL) and *n*-decanol (13.5 mM), under gentle stirring at exactly 25 °C. Aged 1–2 nm gold seeds (18 mL) were then introduced under stirring. The reaction mixture was left undisturbed at 25 °C for 4 h and resulted in a dark gray solution with a longitudinal plasmon resonance peak at ∼725–735 nm. The resulting seeds were purified by centrifugation (12,000 rpm, 60 min) and resuspended in CTAB (10 mM). The final gold concentration was adjusted to 4.65 mM.

Finally, the AuNRs with tunable LSPR bands were synthesized by sequentially adding AgNO_3_ (0.01 M, 200 μL), HAuCl_4_ (0.05 M, 100 μL), and ascorbic acid (0.1 M, 80 μL) to a solution containing CTAB (50 mM, 10 mL) and *n*-decanol (11 mM) at 28 °C. The reaction was initiated by adding HCl (1 M, variable volumes listed in table below) and small anisotropic seeds created in step 2 (50 μL, V_f_: 16 × 10^3^ nm^3^). The LSPR tuning was achieved by controlling the volumes of HCl and seed suspension. The resulting AuNRs exhibited LSPR peaks ranging from 600 to 1000 nm, optimized for applications in the NIR-I region Table 1.

**Table 1:** 

LSPR (nm)	*T* (°C)	AgNO_3_ (μL)	[HAuCl_4_]	ascorbic acid	HCl (μL)
**650**	28	200	100	80	50
**700**	28	200	100	80	100
**750**	28	200	100	80	200
**800**	28	200	100	80	300
**850**	28	200	100	80	400
**900**	28	200	100	80	500
**950**	28	200	100	80	600

### Hybrid Silica-Coating and PEGylation of the AuNRs

A coprecursor mesoporous silica coating protocol was developed built upon the existing protocol by Gorelikov and Matsuura.^66^ A combination of TMOS and TEOS was used to balance hydrolysis and condensation rates, enabling rapid shell initiation and controlled silica growth. This dual-silane approach minimized off-target condensation and promoted uniform coating on nanoparticle surfaces. Briefly, NaOH (1 M, 10 μL) was added to the AuNR solution (10 mL) under constant stirring, followed by a single injection of TMOS (12 μL). After a 10 min interval, successive injections of TEOS (12 μL each) at 30 min intervals, depending on the AuNR1–4. The reaction was allowed to proceed for ∼20 h at room temperature to facilitate the formation of a uniform silica shell around the AuNRs. The resulting silica-coated AuNRs (S-AuNRs) were washed by centrifugation (9000 rpm, 10 min, 6 times) and redispersed in methanol. The final S-AuNRs were redispersed in 95% Ethanol. PEGylation was carried out by incubating the S-AuNRs with an excess of PEG 5 K (1 mL, 10 mM) overnight. Postincubation, the PEGylated particles were washed by centrifugation (9000 rpm, 10 min, 4 times) to remove unbound PEG. The resulting PEGylated silica-coated AuNRs (PS-AuNRs) were redispersed in DI water for subsequent use.

### Characterization of PS-AuNRs

The optical properties of the PS-AuNRs were analyzed using a UV–Vis–NIR spectrophotometer (Evo 220, Thermo Fisher Scientific), recording absorption spectra from 400 to 1100 nm. TEM (HT 7700, Hitachi) was employed to assess the size, morphology, and dispersion state of the nanoparticles. TEM samples were prepared by drop-casting 10 μL of the nanoparticle solution onto a copper mesh grid, followed by air drying overnight. TEM images were analyzed using Gatan Digital Micrograph software. Quantitative measurements of nanoparticle dimensions, including length, width, and aspect ratio, were conducted by analyzing over 100 individual nanoparticles to ensure statistical reliability. Solutions and colorimetric images were analyzed over multiple days to evaluate the colloidal stability of the AuNRs.

### PA Imaging of AuNRs and PS-AuNRs

PA imaging and characterization of the nanorods were conducted using a tube phantom (inner diameter: 3/16 in., outer diameter: 5/16 in.; BD INTRAMEDIC Polyethylene Tubing PE#160) secured within a custom 3D-printed housing. The tubes were filled with 40 μL of each nanorod solution for analysis. US/PA images were acquired using an LZ250 linear array transducer (256 elements, 21 MHz center frequency) paired with the Vevo LAZR system (FujiFilm VisualSonics). Spectroscopic PA imaging was performed over a wavelength range of 680 to 970 nm with 2 nm intervals. Background subtraction was conducted using the PA intensity of a DI water negative control at corresponding wavelengths and laser fluences to ensure accurate signal quantification. PA intensities were analyzed using VevoLAB 5.7.0 software (FujiFilm VisualSonics). For 3D imaging, samples were scanned at specified wavelengths (690, 770, 870, and 940 nm) in 3D mode over a 10.21 mm range with 0.152 mm step intervals. Final tube phantom images were reconstructed in the coronal plane using maximum intensity projection (MIP). Linearity testing was conducted on AuNR 1, 2, 3, and 4 at 4 different concentrations (12.5%, 25%, 50%, and 100%) to generate individual dose–response curves. Crosstalk was assessed at four representative wavelengths (690, 770, 870, and 940 nm) corresponding to the peak absorbance of each agent. At each wavelength the percent signal contribution from nontarget agents relative to the target agent was calculated. Spectral overlap between agents was quantified using the normalized overlap integral. Spectra were baseline-subtracted and peak-normalized. The integral was evaluated numerically using the trapezoidal rule. This metric reflects the fractional overlap of agent *j* with respect to agent *i*.

$$Overlap_{ij}=\frac{\int \min\left(S_{i}\left(\lambda \right),S_{j}\left(\lambda \right)\right)d\lambda }{\int S_{i}\left(\lambda \right)d\lambda }$$

### Photostability and Linearity Characterization of AuNRs and PS-AuNRs

The photostability of PS-AuNRs was evaluated through evaluation of the PA signal after extended pulsed laser exposure. The PA stability of the nanorods was assessed using the tube phantom setup described previously. Initial PA spectra were recorded over a wavelength range of 680 to 970 nm with a 2 nm interval using the Vevo LAZR system. Samples were then irradiated with >5000 laser pulses (7 ns pulse duration, 10 Hz pulse repetition rate) at wavelengths 690, 770, 870, and 940 nm each with the changes in PA signal being monitored. Laser fluence was applied across a range of 13.75 ± 0.43 mJ/cm^2^ to 17.56 ± 0.65 mJ/cm^2^. After irradiation, final PA spectra were recorded over the same wavelength range to evaluate changes in optical performance. Linearity measurements were conducted on all 4 PS-AuNRs at progressively increasing OD = 0.125, 0.25, 0.50, and 1.00 in the tube phantom.

### Multiplex Algorithm and Analytics

A custom spectral unmixing algorithm was developed to accurately quantify the relative contributions of each spectrally distinct PS-AuNR contrast agent within multiplexed PA imaging. The algorithm utilized NNLS regression, applied to spectroscopic PA data collected from known PS-AuNR mixtures, enabling precise deconvolution of overlapping absorbance spectra. Known absorbance spectra for each individual contrast agent were acquired using either the UV–Vis–NIR spectrophotometer or the Vevo LAZR and stored in a reference data set. Mixed spectra corresponding to different ratios of PS-AuNRs were obtained under identical measurement conditions. All spectra were smoothed using a Savitzky–Golay filter (3rd-order polynomial, frame size = 15) to reduce noise while preserving spectral shape. The unmixing algorithm was implemented in MATLAB to solve a constrained optimization problem where the measured mixed spectra (*S*_*m*_) were expressed as a linear combination of the known contrast agent spectra (*S*_*k*_)­

$$S_{m}=S_{k}C+\varepsilon$$

where *C* represents the unknown contributions of each contrast agent, and ε accounts for residual error. The NNLS solver was applied to constrain C to non-negative values, ensuring physically meaningful solutions. To assess the accuracy of spectral unmixing, reconstructed spectra were generated from the computed contributions and compared to the original mixed spectra. Mean absolute error (MAE) was calculated between algorithm-estimated and ground-truth contributions obtained from known mixture compositions.

$$MAE=\frac{1}{N}\sum_{i=1}^{N}|C_{i}^{true}-C_{i}^{\hat{estimated}}$$

$$C_{i}^{true}=ground\;truth\;contributions;C_{i}^{estimated}=estimated\;contributions$$

Absolute error distributions were analyzed across individual mixtures and contrast agents. After validating the algorithm with UV–Vis–NIR data, it was applied to PA imaging data sets acquired from multiplexed PS-AuNR solutions. Spectroscopic PA data (680–970 nm in 2 nm increments) were processed using the unmixing algorithm, enabling quantitative deconvolution of multiple contrast agents within complex mixtures. The final output included spatially resolved PA maps highlighting the relative distribution of each PS-AuNR population.

### US/PA Multiplexing Evaluation of PS-AuNR Mixtures

31 unique mixtures were prepared using varying ratios of four distinct PS-AuNRs (PS-AuNR1 to PS-AuNR4). The ground truth contribution data for the ratios in each mixture are detailed in Table S1. The UV–Vis–NIR spectra of all mixtures were recorded using a spectrophotometer (Evo 220, Thermo Fisher Scientific), and these data were used to characterize the contributions of each contrast agent using the multiplex algorithm as described above. The algorithm was fine-tuned to minimize error and improve accuracy in deconvoluting spectral data. Spectroscopic US/PA imaging was performed on all 31 mixtures (680–970 nm, 2 nm intervals). The updated multiplex algorithm was applied to the PA imaging data to unmix the contributions of the four contrast agents across the different mixtures. Reconstructed spectra were generated for each mixture, the ground truth and the estimated contributions were compared, and the MAE for the estimated contributions of each mixture was calculated relative to the ground truth data. MAE % per mixture and per agent were calculated. To explore the impact of design parameters, MAE % was plotted as a function of: (1) number of agents per mixture, to assess the effect of spectral complexity, (2) minimum spectral spacing (in nm) between agents in a mixture, calculated as the smallest pairwise difference in peak wavelengths, and (3) ground truth contribution of each agent, to test whether abundance influenced prediction error. Additionally, agent-wise MAEs were aggregated across all mixtures to reveal agent-specific unmixing performance. All analysis was conducted using MATLAB R2020a and the visualizations were implemented using GraphPad Prism, with error bars reflecting standard deviation where applicable.

### Cell Culture

For in vitro toxicity experiments, Human adipose derived mesenchymal stromal/stem cells (hADMSCs, Lonza Biosciences, Basel, Switzerland) were cultured in α-minimum essential medium (α-MEM) supplemented with 10% fetal bovine serum, and 1% penicillin–streptomycin. Cells were incubated under standard conditions at 37 °C and 5% CO_2_ in a humidified incubator (Heracell VIOS 160I, Fisher Scientific). Culture media was replaced every 3 days and cells were passaged at ∼80–90% confluence. For passaging, hADMSCs were detached using 0.05% Trypsin–EDTA, neutralized with α-MEM, centrifuged, counted with a hemocytometer, and finally seeded onto fresh cell-culture T75 or T175 flasks at approximately 5000 cells/cm^2^. Only hADMSCs between passages P4 and P8 were used.

### In Vitro Cytotoxicity Evaluation of PS-AuNRs

hADMSCs were seeded in 96-well plates at 5000 cells per well and cultured for 24 h at 37 °C under 5% CO_2_. Cells were then incubated with PS-AuNR1, PS-AuNR2, PS-AuNR3, or PS-AuNR4 over a concentration range corresponding to 0, 0.5, 1, 2, 4, 8, and 16 OD (*n* = 3 independent replicates per condition). After overnight incubation, cells were washed thoroughly with PBS to remove unbound nanoparticles. MTT solution (0.5 mg/mL) was then added to each well and incubated for 4 h. The resulting formazan crystals were dissolved in DMSO, and absorbance was measured at 590 nm using a plate reader (Synergy HT, BioTek). Cell viability was quantified relative to the nanoparticle-free control. Statistical comparisons were performed using one-way ANOVA with Dunnett’s multiple-comparisons test against the nanoparticle-free control for each formulation.

### US/PA Multiplexing Evaluation in Whole-Blood Background

To test robustness against strong endogenous absorbers, PS-AuNRs1–4 were reprepared in human blood (obtained from the discard blood from a hematology lab (Georgia Institute of Technology Institutional Review Board [IRB] No. H23498) at the same total PS-AuNR concentration. UV–Vis–NIR spectroscopy was performed for blood and the blood PS-AuNR mixtures (400–1100 nm). Samples were loaded into the tube phantom and imaged under matching spectroscopic conditions (Vevo LAZR, 532 nm, 680–970 nm, 2 nm steps). PA spectra and PA signal were evaluated at the different wavelengths to assess PA performance of PS-AuNRs in the present of blood.

### Animal Studies

All animal procedures were approved by the Institutional Animal Care and Use Committee (IACUC) at the Georgia Institute of Technology under protocol A100284 in accordance with federal guidelines for the care and use of laboratory animals. Female nu/J mice (10–14 weeks old) were used for all the studies. Per IACUC guidelines, animals were not imaged on consecutive days, and each imaging session was limited to a maximum of 4 h to minimize stress.

### In Vivo PA Multiplexing of Subcutaneous Injections

For subcutaneous injections, PS-AuNR solution was prepared by centrifugation to remove DI water, followed by resuspension in sterile PBS. To ensure sterility, the solution was kept under UV-irradiation for 30 min. Mice were anesthetized using isoflurane gas (5% induction, 2% maintenance) mixed with medical-grade oxygen (0.6–0.8 L/min). Injections containing known mixtures of PS-AuNR1 to PS-AuNR4 (Table S3), which were prepared in Matrigel, (1:1, final OD = 5) were subcutaneously administered on the dorsal side of the mouse (*n* = 5 biological replicates per experimental condition). To minimize animal use in the study, two sets of experimental conditions (4 subcutaneous injections each) were performed, one on the right and one on the left dorsal flank, thus reducing the number of mice to half. The injection site was sterilized with an alcohol swab prior and post injection to minimize the risk of infection. Injections containing saline only and Matrigel with saline were used as negative controls (*n* = 1 each). US/PA imaging was performed immediately following the subcutaneous injection. During imaging the anesthetized mice were placed on a heated, motorized translational stage equipped with an anesthesia cone to maintain stable respiration and body temperature throughout the procedure. In vivo subcutaneous US/PA images were acquired (LZ250 transducer, 21 MHz, Vevo LAZR). PA spectral scans (680–970 nm, 2 nm increments) and 3D PA imaging were conducted as described previously. For 3D imaging, the transducer was translated along the dorsal plane of the mouse using a motorized stage, acquiring data at 0.152 mm intervals. Images were processed and analyzed using both MATLAB and VevoLAB software (v5.7.0, Fujifilm VisualSonics).

### In Vivo Multiplex US/PA Imaging and Data Analysis

US/PA spectroscopy data sets were batch-processed using a custom MATLAB pipeline. To enhance signal fidelity, preprocessing was applied in multiple stages. First, a global threshold was computed as 5% of the maximum PA signal intensity in each data set. Next, a Savitzky–Golay filter (polynomial order = 3, frame size = 15) was applied to the spectral dimension of each pixel in the thresholded PA stack to smooth high-frequency noise while preserving spectral features. Pixels with insufficient nonzero spectral points (<15) were left unsmoothed. Known spectra for each contrast agent (PS-AuNR1–4) were imported from a reference spreadsheet (680–970 nm, 2 nm increments). Spectra were baseline-subtracted to reduce tissue background and system-level wavelength dependence and column-normalized by their maximum values. A same-sized background ROI was placed in nearby tissue at similar depth but outside the injection zone, and the resulting depth-matched background spectrum was subtracted from the experimental ROI prior to downstream analysis. This local background subtraction was applied as a baseline-correction step for spectral interpretation and unmixing. For each pixel in the smoothed 3D PA data set, NNLS spectral unmixing was performed to solve for agent-specific contribution weights. This generated spatially resolved contribution maps for each agent. Unmixed agent-specific contribution maps were arranged into subplots with agent-wise color bars. To compare raw PA signal with multiplexed outputs, MIP images were generated from the smoothed PA stack and displayed alongside multiplexed images using two visualization strategies: (1) dominant agent mapping, where each pixel was color-coded according to the agent with the highest unmixed contribution; and (2) additive merging, where RGB overlays were computed by summing agent-specific color channels, weighted by their normalized contribution values. For display purposes only, a single fixed intensity threshold and common visualization scaling were applied across agents and data sets to suppress low-level background and maintain direct visual comparability between panels. Dynamic or image-specific thresholding was not used, as it would introduce panel-dependent tuning and reduce consistency in side-by-side comparisons. Custom colormaps were used for each agent. Pixel intensities were rescaled using minimum and maximum bounds (min = 0, max = 3800) to standardize visual outputs across data sets. Quantitative graphs comparing the estimated contributions of each contrast agent with the ground truth data were created (*n* = 5). These visualizations provided a comprehensive assessment of the accuracy and spatial resolution of the multiplex imaging technique.

### Quantitative Accuracy Assessment of Multiplexed Unmixing Using Spatial and Intensity-Based Metrics

A two-part analysis pipeline assessing both spatial localization and signal quantification accuracy for each contrast agent was implemented. For each of the four PS-AuNR agents, manual region-of-interest (ROI) masks were drawn within the binary MIP to define ground truth injection zones. Spatial detection masks were then created for each unmixed contribution map by thresholding at 5% of the agent’s maximum signal. For each agent, three performance metrics: sensitivity, specificity, precision were computed using pixel-wise comparison between the detection and ground truth masks. Definitions were as follows. Here, TP, FP, FN, and TN represent the number of true positive, false positive, false negative, and true negative pixels, respectively. These metrics evaluate spatial localization in a binary manner, that is, whether a pixel is correctly assigned as present or absent for a given agent.

$$Sensitivity=\frac{TP}{\left(TP+FN\right)}$$

$$Specificity=\frac{TN}{\left(TN+FP\right)}$$

$$Precision=\frac{TP}{\left(TP+FP\right)}$$

To complement binary classification metrics, we performed a parallel analysis based on signal intensities rather than pixel presence using the same ROIs defined above. To further characterize off-target detection, we report normalized signal leakage fractions, defined as the fraction of signal erroneously assigned to FP or FN zones relative to the total signal. Unlike sensitivity and specificity, this conservative metric captures even subtle spectral bleed-through that may be missed by spatial analysis.

$$Signal\;Leakage\;Ratio\left(SLR\right)=\frac{FP_{Intensity}}{{\left(TP+FP\right)}_{Intensity}}$$

### Built-In VevoLAB Multiplexer Analysis in the Presence of Endogenous Absorbers

To evaluate the compatibility and practical translational accessibility of the PS-AuNR multiplexing platform within a standard commercial workflow, selected spectroscopic PA data sets were also analyzed using the built-in VevoLAB multiplexer module (version 5.7.0, FUJIFILM VisualSonics). The same representative subcutaneous data sets used for the custom NNLS-based analysis were reanalyzed in VevoLAB using the corresponding PS-AuNR basis spectra. These reference spectra were uploaded into the software as custom contrast channels, and spectral deconvolution was performed using the platform’s native unmixing and display tools. Because the VevoLAB multiplexer supports a maximum of five channels, the four PS-AuNR channels could be analyzed either alone or with the addition of one endogenous hemoglobin channel at a time, either deoxyhemoglobin or oxyhemoglobin. VevoLAB visualizes multiplexed channels as sequential layered overlays, the apparent prominence of a given channel depends in part on its display order. For consistency, multiplexed renderings were generated using a fixed channel display order in which PS-AuNR1, PS-AuNR2, PS-AuNR3, and PS-AuNR4 were sequentially layered. When deoxyhemoglobin or oxyhemoglobin was included as an additional channel, it was placed at the top of the rendering stack to facilitate visual assessment of potential endogenous background interference. Additional renderings were also generated with alternative layer orders to examine the effect of sequential overlay order on visual interpretation. In separate qualitative tests, the display gain of the hemoglobin channels was increased to assess whether endogenous signal could obscure or confound interpretation of the PS-AuNR signals under exaggerated visualization conditions.

### Statistics

For in vitro experiments, at least three technical replicates were run for each set of experimental conditions. For in vivo experiments, at least five independent biological replicates (animals) per group were used. Different synthesis batches of PS-AuNRs were used in a randomized pattern, also synthesized by different operators. For experiments involving two groups, control and experimental samples were compared by an unpaired Student’s *t*-test since each sample replicate was independent of every other sample replicate, and the replicate data arose from a randomized process. A Jarque-Bera goodness-of-fit test was conducted on all data to confirm that the skewness and kurtosis matched a normal distribution prior to the Student’s *t*-test. For experiments involving 3 or more groups, experimental conditions were compared using one-way or two-way ANOVA with replicated measurements followed by a post hoc Tukey test (95% confidence interval) to calculate effect size and the confidence interval limits (* <0.05, ** <0.01, *** <0.001, **** <0.0001) as appropriate. Unless otherwise noted, all statistical analyses were performed using Microsoft Excel, R, or GraphPad Prism version 8.0.1 (GraphPad Software, La Jolla, CA, USA).

## Supplementary Material



## Data Availability

The authors confirm that the data supporting the findings of this study are available within the article and its Supporting Information. Any additional data is available from the corresponding author upon request. The code of the NNLS multiplexing model and the analytics pipeline is available from the corresponding author on reasonable request.

## Notes

Schematic representations of three AuNR synthesis methods (one-step seedless, two-step seed-mediated, and three-step tunable), as well as conventional and updated hybrid silica coating protocols. Simulated electric field distributions, TEM images, DLS/PDI/zeta-potential measurements, and batch-to-batch UV–Vis–NIR/TEM analyses characterize size, morphology, surface properties, spectral tunability, and synthesis reproducibility of PS-AuNR1–4. PA linearity and photostability measurements in tube phantoms validate concentration-dependent signal response and spectral stability under prolonged laser irradiation. In vitro multiplexing results (Mixes 1–31) include representative US/PA images, original versus reconstructed spectra, and estimated versus ground truth contributions. Cytotoxicity studies evaluate PS-AuNR biocompatibility in hADMSCs. UV–Vis and PA spectral analyses demonstrate signal separation of AuNRs from blood, while raw in vivo background measurements assess endogenous PA signal across the NIR-I window. Subcutaneous injection studies include merged multiplexed maps, Matrigel-only and PBS-only negative controls, and spectral unmixing performance across Groups 1–12, VevoLAB-based multiplexing comparisons, layer-order analysis, and unmixing with added oxyhemoglobin and deoxyhemoglobin basis spectra. Analysis of spectral spacing and agent concentration highlights factors influencing unmixing accuracy. Tables S1 and S2 summarize LSPR and PA spectral FWHM values; Tables S3 and S4 detail ground truth and estimated contributions for in vitro mixtures; Tables S5 and S6 provide corresponding data for in vivo subcutaneous injections (PDF)
