# Supplementary material for: Quad-Channel In Vivo Photoacoustic Multiplexing Using Tunable Gold Nanorods
Source: ACS Nano. 2026 May 7;20(21):15133–52. doi: 10.1021/acsnano.5c22519 (PMC13235648; doi:10.1021/acsnano.5c22519)
Supplement: Supplementary file 1 [file nn5c22519_si_001.pdf]

Supporting Information

Quad-Channel In Vivo Photoacoustic Multiplexing Using Tunable Gold Nanorods

Anamik Jhunjhunwala[1], Myeongsoo Kim[1,2], Huyju Mun[1], Brooke Chambliss[1], Jinhwan Kim[3,4], and Stanislav Y. Emelianov[1,2,5,]*

[1] Wallace H. Coulter Department of Biomedical Engineering, Georgia Institute of Technology and Emory University School of Medicine, Atlanta, GA, 30332, USA
[2] Petit Institute for Bioengineering and Bioscience, Georgia Institute of Technology, Atlanta, GA, 30332, USA
[3] Department of Biomedical Engineering, University of California Davis, Davis, CA, 95616, USA
[4] Department of Surgery, University of California Davis, Sacramento, CA, 95616, USA
[5] School of Electrical & Computer Engineering, Georgia Institute of Technology, Atlanta, GA, 30332, USA

* Corresponding author: Stanislav Y. Emelianov
🖂 E-mail: stas@gatech.edu

KEYWORDS: Photoacoustic imaging, Multiplexing, Nanoparticles, Gold nanorods, Silica coating, Spectral Unmixing, *In vivo* Multiplex Imaging

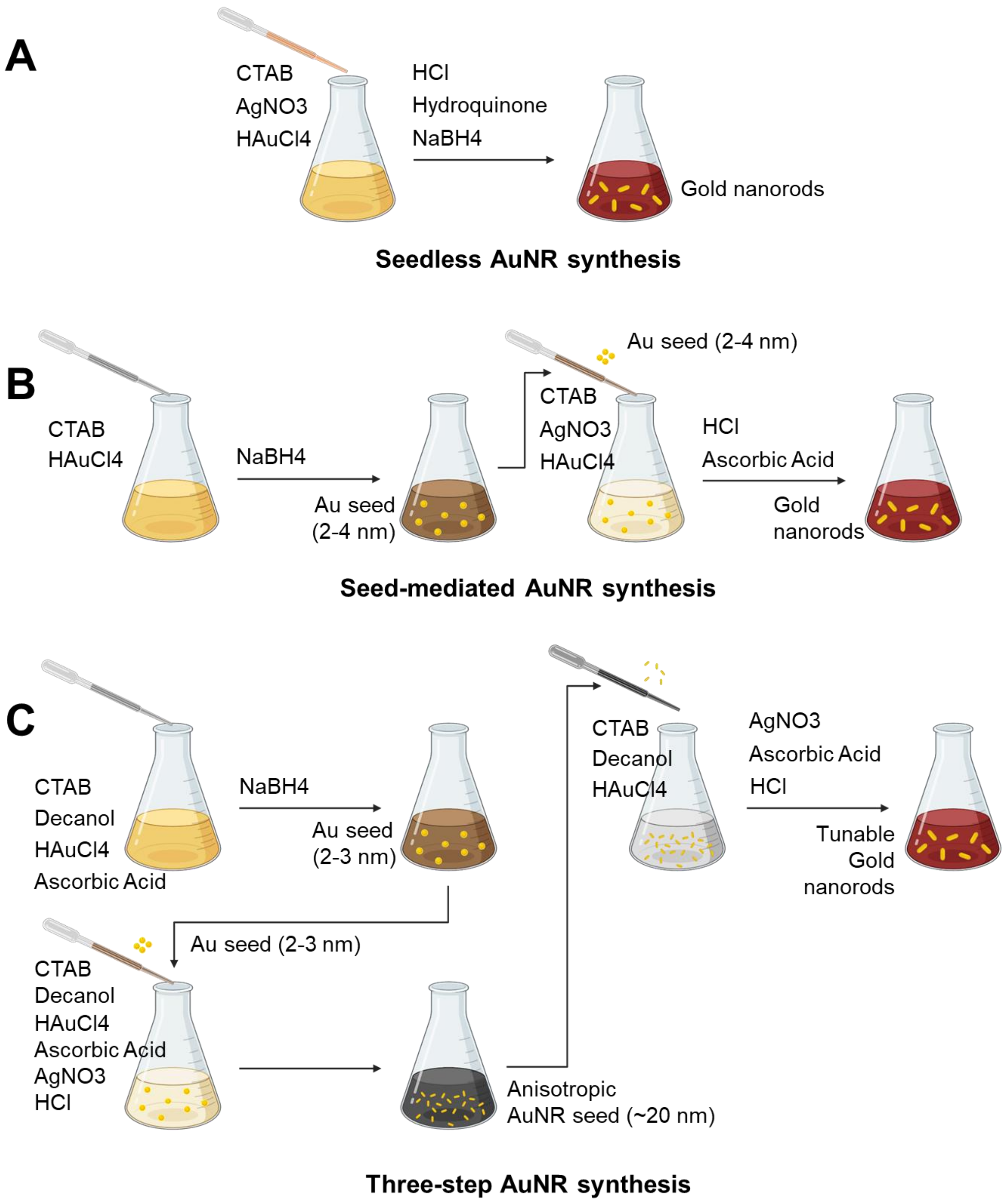

Fig S1. Schematic representations of three different synthesis methods for gold nanorods (AuNRs). A. One-step seedless synthesis, in which AuNRs form directly in a single growth solution without the use of a preformed seed template. **B**. Two-step seed-mediated synthesis, where spherical isotropic gold seeds are first synthesized and then grown into anisotropic AuNRs. **C.** Modified three-step synthesis protocol used in this study: Step 1 produces isotropic spherical gold seeds; Step 2 yields miniature, highly uniform anisotropic AuNRs; and Step 3 enables precise tuning of aspect ratio through controlled elongation.

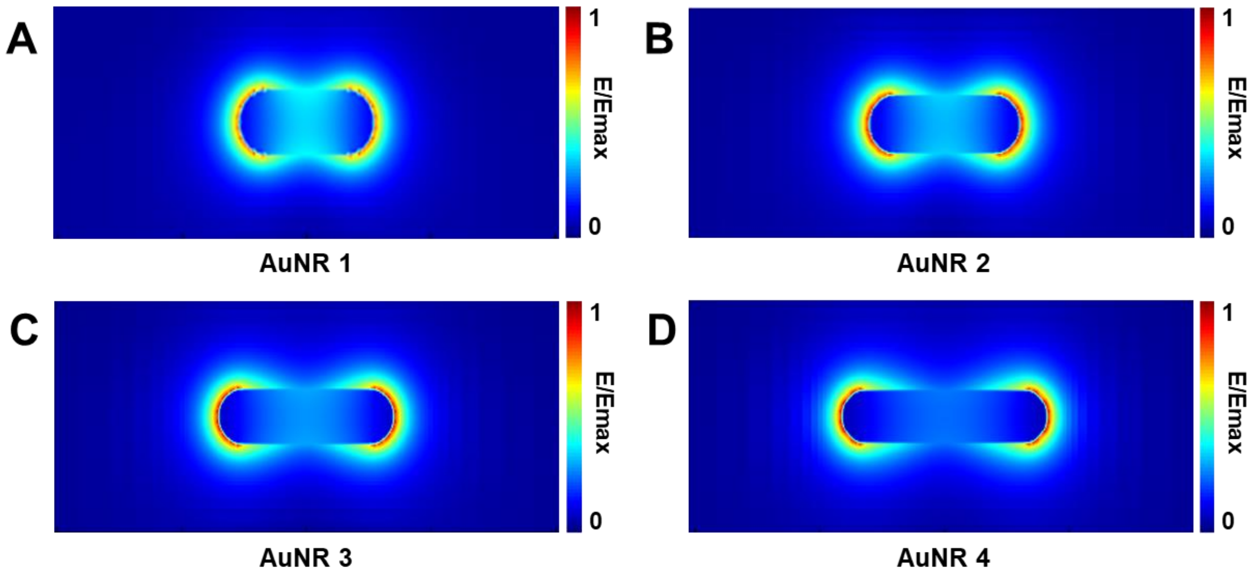

Fig S2. Simulated electric field distributions for gold nanorods with varying aspect ratios. A–D. Finite-difference time-domain (FDTD) simulations of localized electric field distributions for AuNR1 through AuNR4, respectively. Each nanorod exhibits strong electric field enhancement localized at the rod tips upon light excitation. This tip-concentrated field could lead to localized photothermal heating and potential photodamage under prolonged laser illumination. Subsequent silica coating helps mitigate this effect by dissipating heat and stabilizing the nanorod structure.

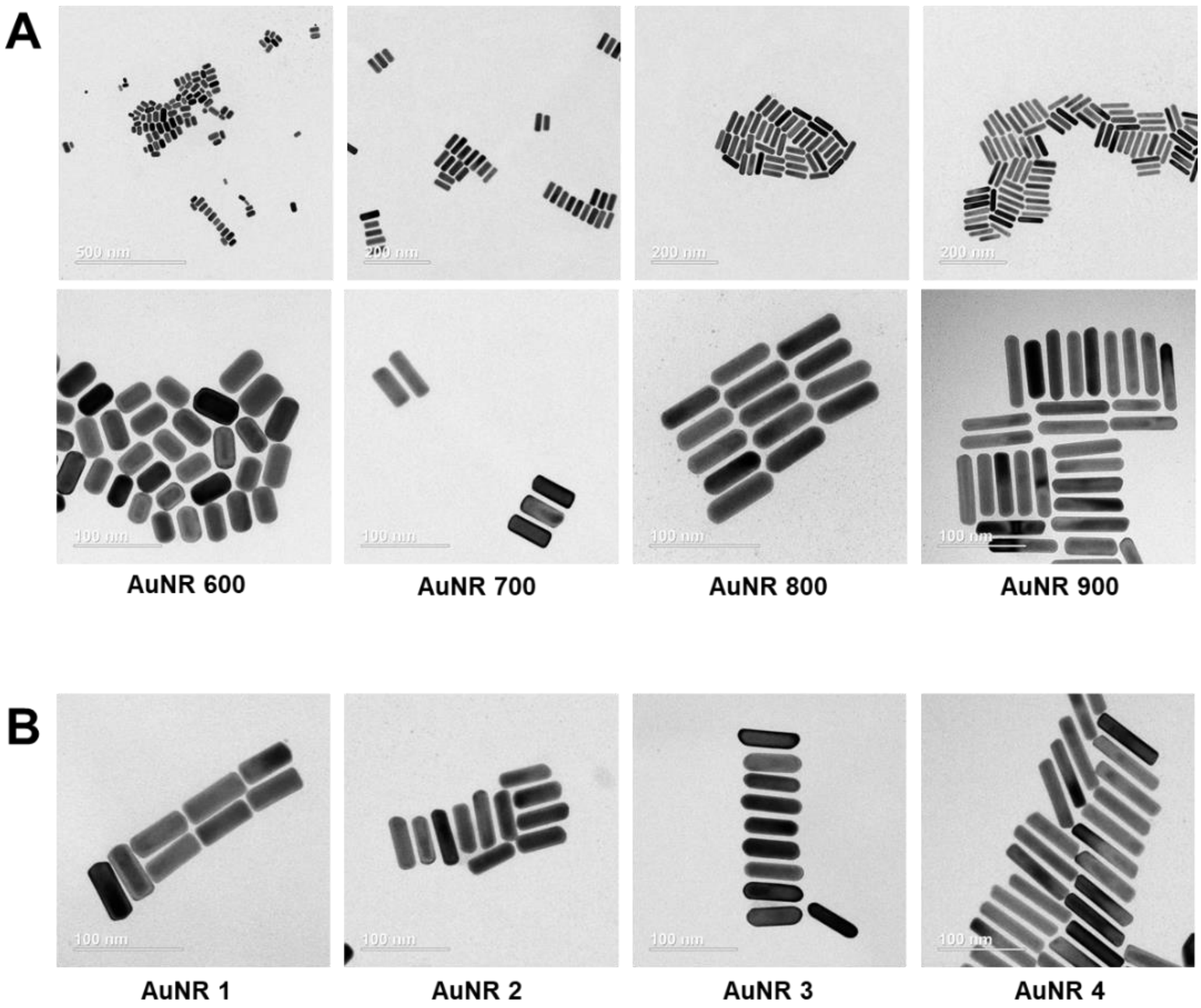

Fig S3. Transmission electron microscopy (TEM) images of gold nanorods synthesized using the three-step protocol. A. Representative TEM images of gold nanorods synthesized to target longitudinal plasmon resonance peaks at approximately 600, 700, 800, and 900 nm, demonstrating the tunability of aspect ratio and uniformity across the spectral range. **B.** Additional TEM images of AuNR1 through AuNR4, highlighting consistent rod morphology and size distribution for the four spectrally distinct nanorod formulations.

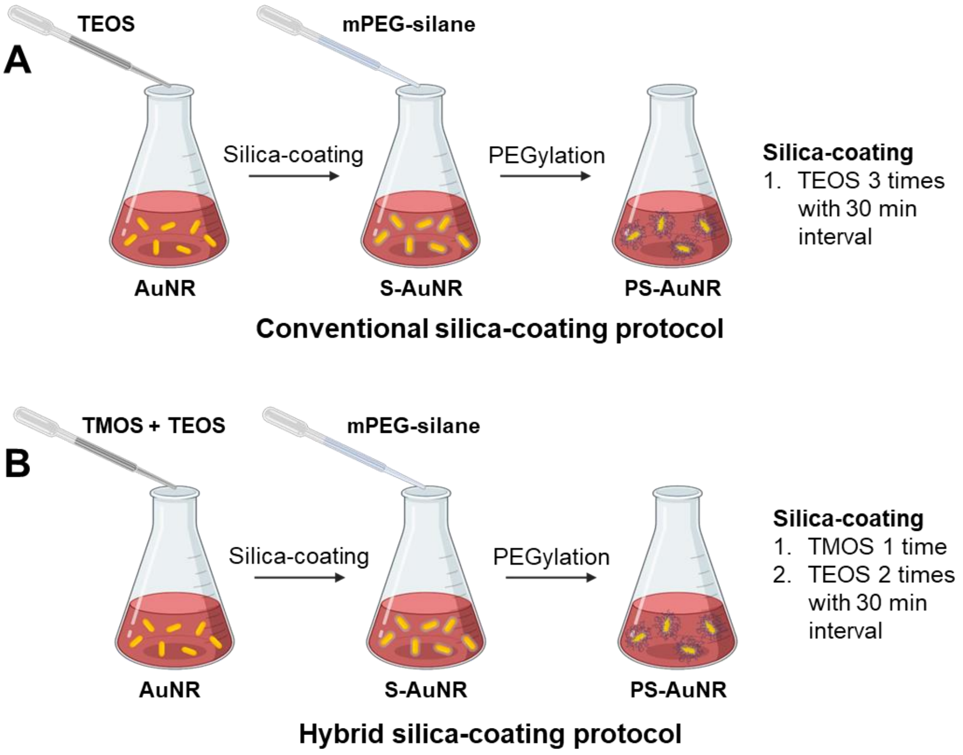

Fig S4. Schematic of conventional and updated silica coating protocols for AuNRs. A. Conventional silica coating method using tetraethyl orthosilicate (TEOS) as the sole silica precursor, followed by PEGylation. **B.** Updated hybrid protocol developed in this study, in which silica growth is initiated with tetramethyl orthosilicate (TMOS) and subsequently continued with TEOS. The coating strategy leverages fast-hydrolyzing TMOS for rapid nucleation and slow-hydrolyzing TEOS for uniform shell growth. This dual-precursor approach balances reaction kinetics, minimizing free silica formation and promoting conformal deposition on curved nanoparticle surfaces. This approach also enables finer control over shell thickness. Followed by PEGylation, this results in PEGylated silica-coated AuNRs (PS-AuNRs) that are geometrically similar in overall size due to fine control over silica-shell thickness, chemically similar due to the identical silica and the PEG coating yet remain spectrally distinct due to the different aspect ratios of the AuNRs.

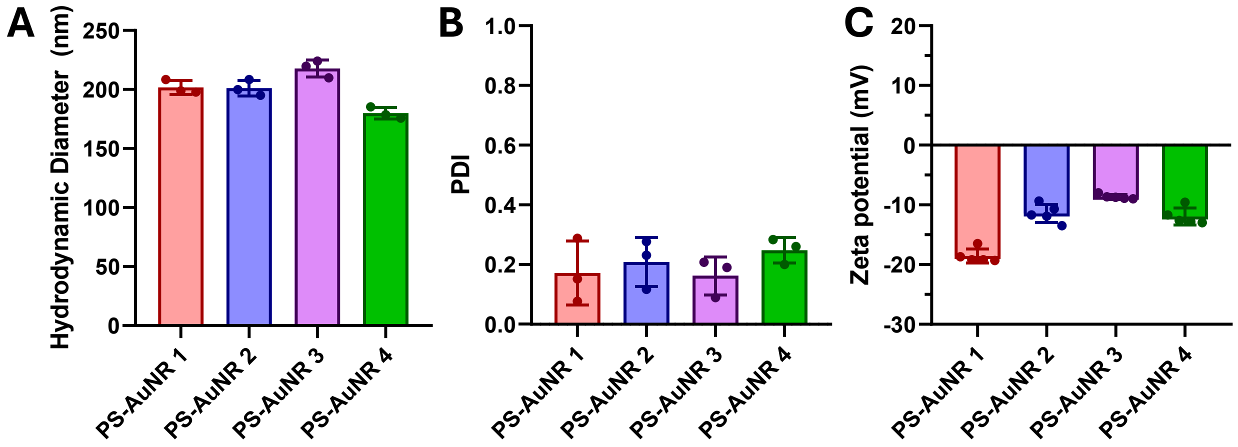

Fig S5. Dynamic light scattering (DLS) characterization of PS-AuNR1-4. (A) Hydrodynamic diameter measured by DLS for PS-AuNR1-4. (B) Polydispersity index (PDI) for PS-AuNR1–4. (C) Zeta potential for PS-AuNR1-4. Together, these measurements depict similar effective hydrodynamic size, size distribution, and closely matched surface charge following silica coating and PEGylation. Data represent independent replicate measurements across batches.

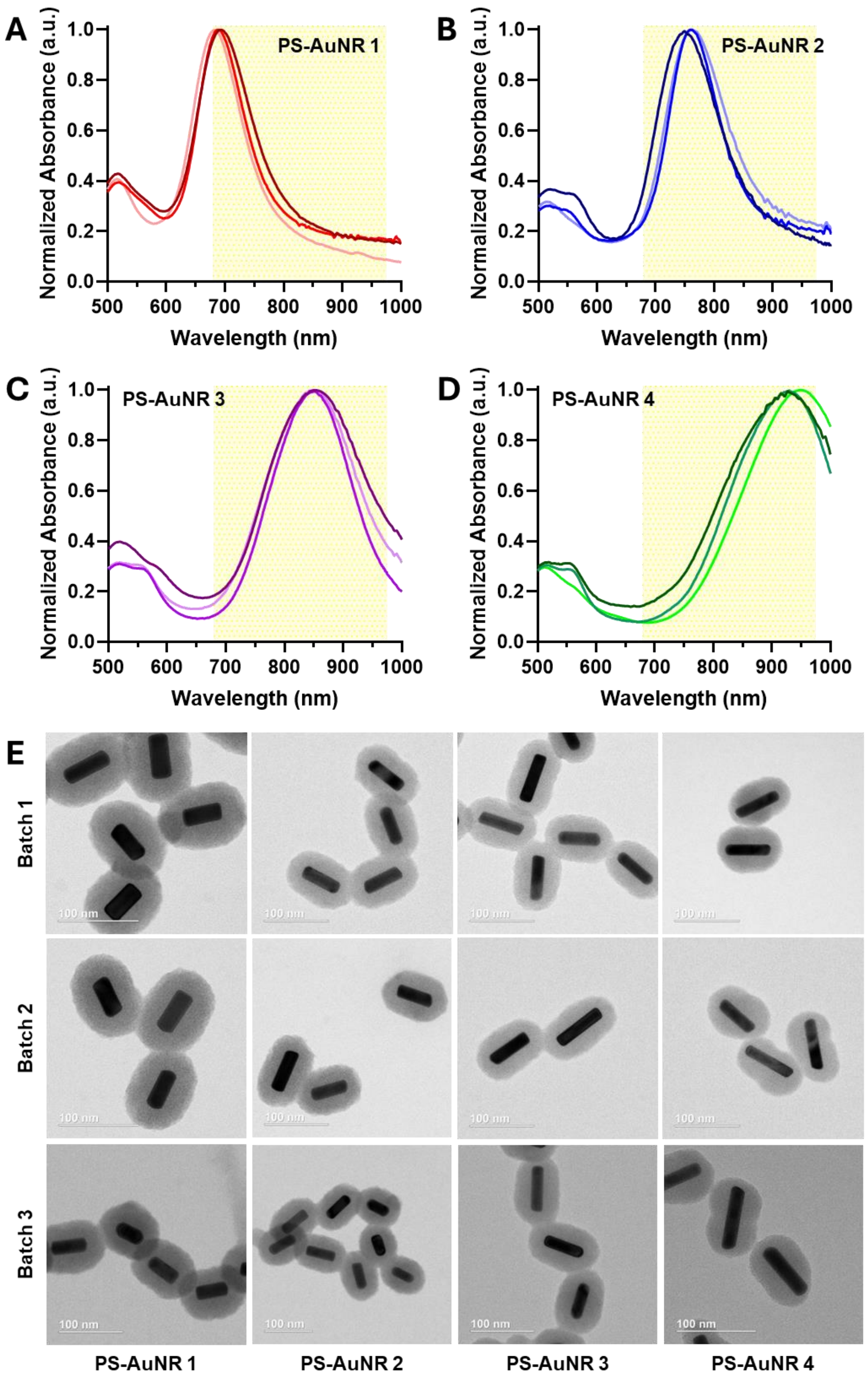

Fig S6. Batch-to-batch reproducibility of PS-AuNR1–4 across three independent synthesis batches. (A–D) UV–Vis–NIR absorbance spectra of PS-AuNR1, PS-AuNR2, PS-AuNR3, and PS-AuNR4, respectively, synthesized in three independent batches, showing closely matched longitudinal LSPR peak positions and overall spectral profiles across batches. The measured longitudinal LSPR peaks were 692, 690, and 687 nm for PS-AuNR1; 754, 745, and 749 nm for PS-AuNR2; 849, 851, and 849 nm for PS-AuNR3; and 925, 931, and 914 nm for PS-AuNR4. (E) Representative TEM micrographs of PS-AuNR1–4 from the same three independent batches, demonstrating consistent nanorod morphology across syntheses.

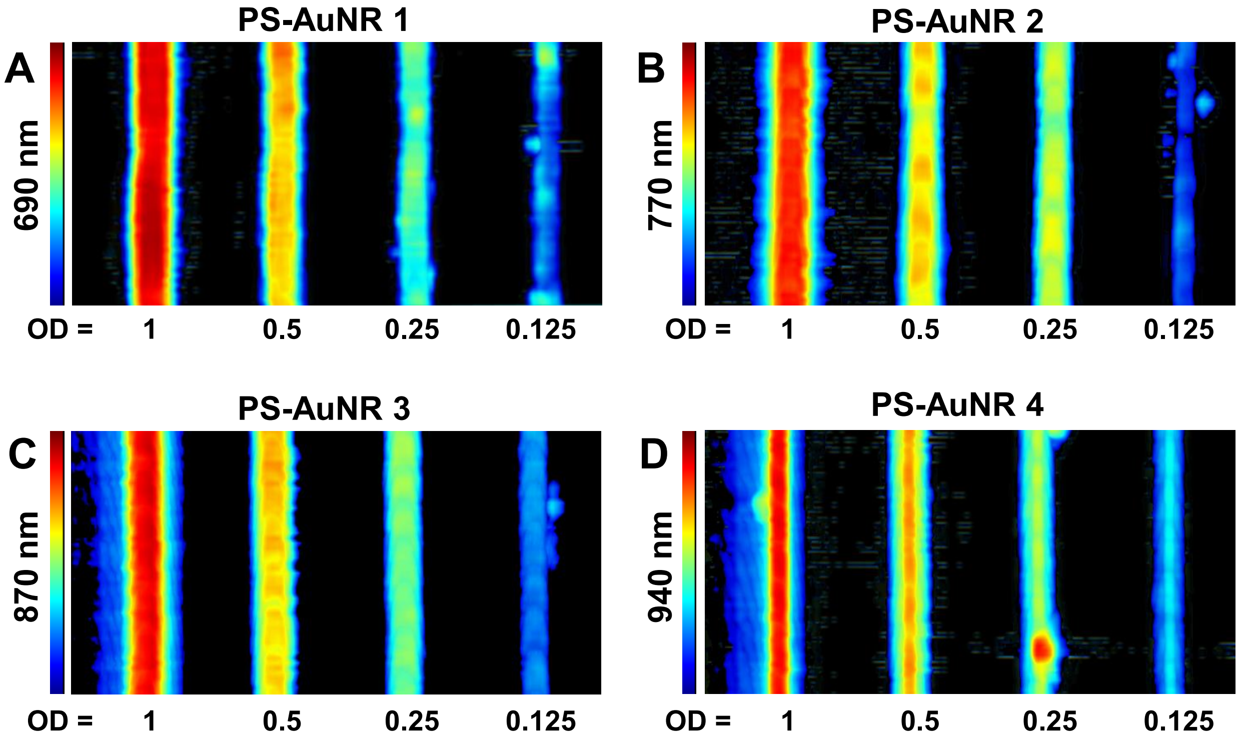

Fig S7. Photoacoustic linearity measurements of AuNR1–4 in tube phantoms. A–D. PA images of tube phantoms containing PS-AuNR1 (A), PS-AuNR2 (B), PS-AuNR3 (C), and PS-AuNR4 (D), acquired at their respective peak wavelengths: 690, 770, 870, and 940 nm. Each panel includes four tubes corresponding to optical densities (OD) of 1.0, 0.5, 0.25, and 0.125. For all nanorods, PA signal intensity decreases proportionally with decreasing OD, demonstrating strong linearity between nanoparticle concentration and photoacoustic response.

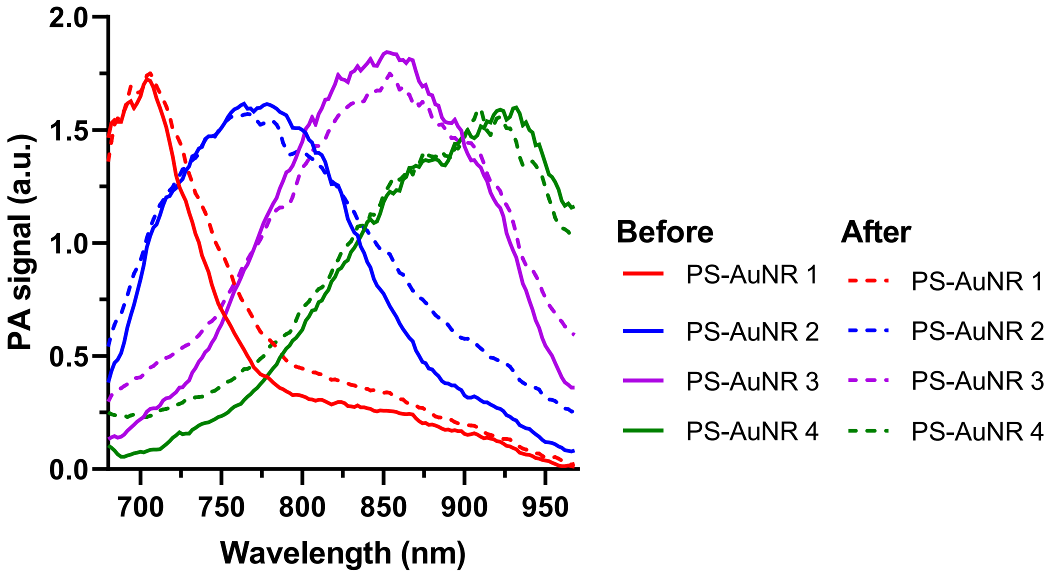

Fig S8. PA spectra of PS-AuNR1–4 before and after extended laser irradiation. PA spectra acquired before and after the photostability experiment for PS-AuNR1, PS-AuNR2, PS-AuNR3, and PS-AuNR4 under the same extended irradiation conditions used in Figure 2F. Laser irradiation was sequentially performed at 680, 770, 870, and 940 nm (>5000 pulses each). Across all four formulations, the spectral profiles and peak positions remained closely matched after irradiation, supporting spectral stability under prolonged pulsed laser exposure.

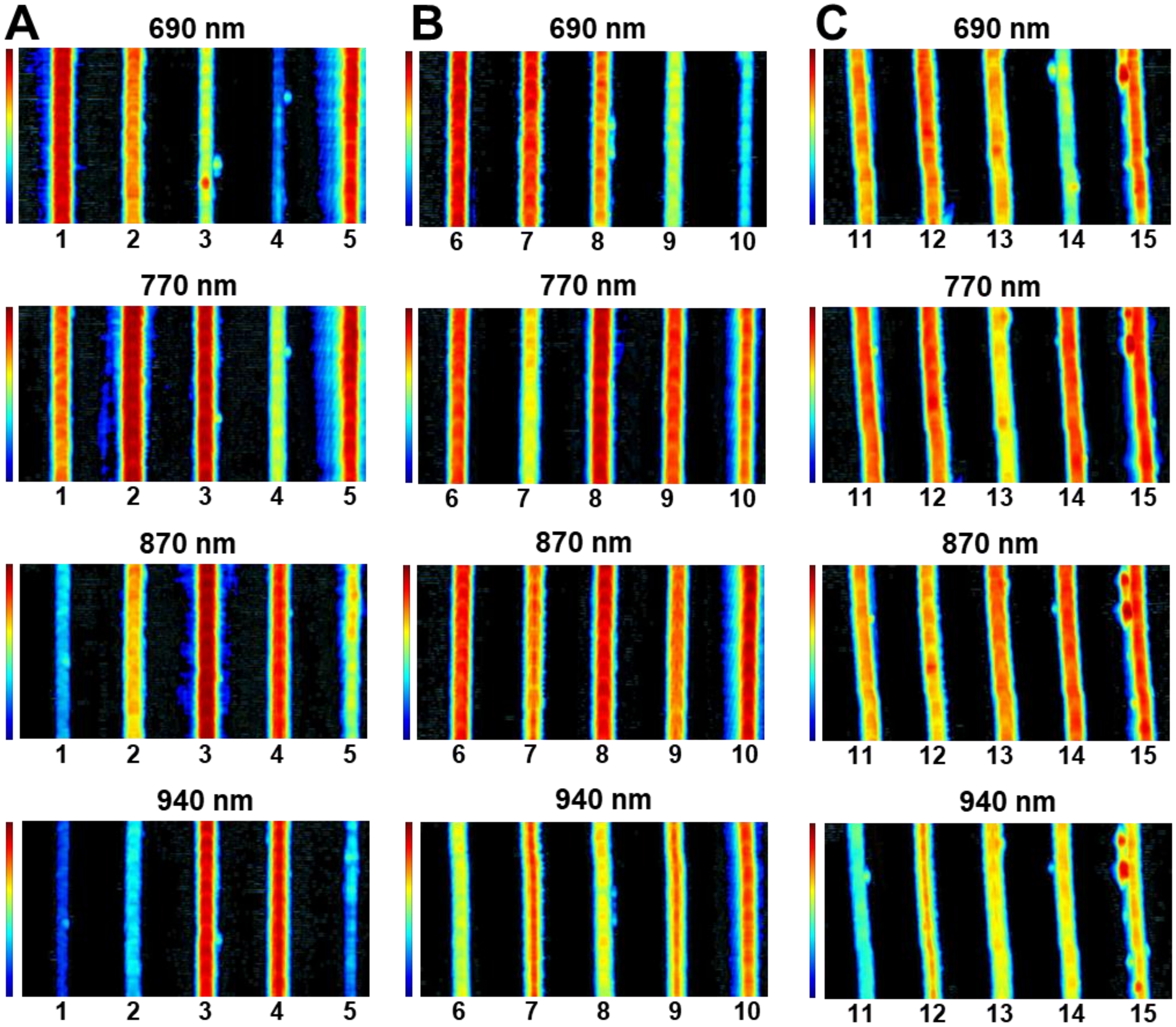

Fig S9. Representative US/PA images of mixtures 1–15 in the *in vitro* phantom multiplexing study. A–C. PA images of mixtures 1–15, spanning single-agent, binary, ternary, and quaternary formulations. Each panel displays five mixtures: A shows Mixes 1–5, B shows Mixes 6–10, and C shows Mixes 11–15. For each mixture, four images are shown corresponding to excitation wavelengths of 690 nm (top row), 770 nm (middle top), 870 nm (middle bottom), and 940 nm (bottom row), representing the peak wavelengths of AuNR1–4. All images were acquired under identical conditions using a custom-build tube phantom setup.

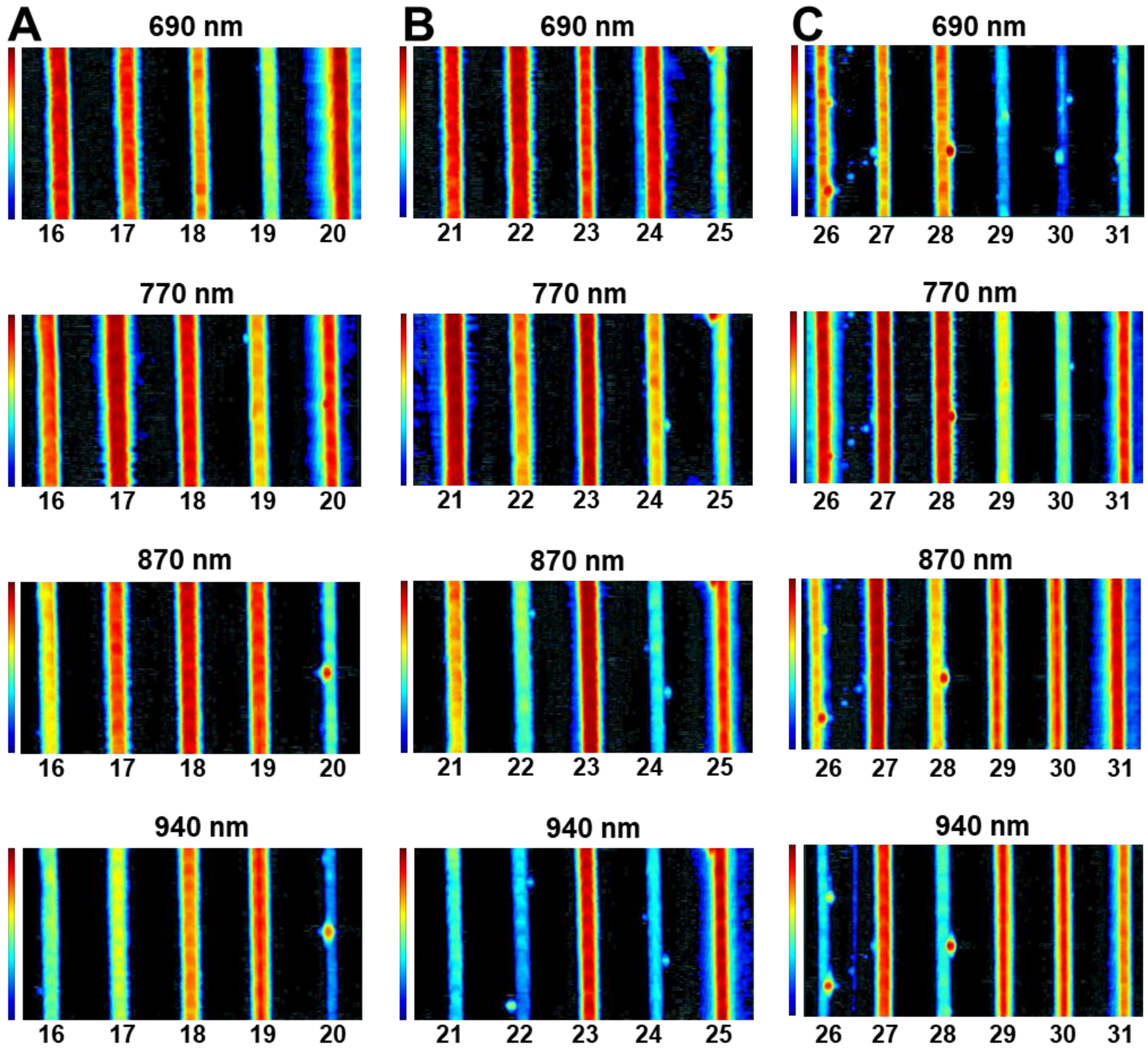

Fig S10. Representative US/PA images of boundary mixtures (Mixes 16–31) in the in vitro phantom multiplexing study. A–C. US/PA images of the remaining 16 mixtures, which include boundary binary and boundary quaternary cases. A shows Mixes 16–20, B shows Mixes 21–25, and C shows Mixes 26–31. Each panel follows the same imaging format as in Supplementary Figure S6, with rows corresponding to 690, 770, 870, and 940 nm excitation wavelengths. These images demonstrate spectral response patterns for mixtures with skewed contribution ratios across the four AuNR types.

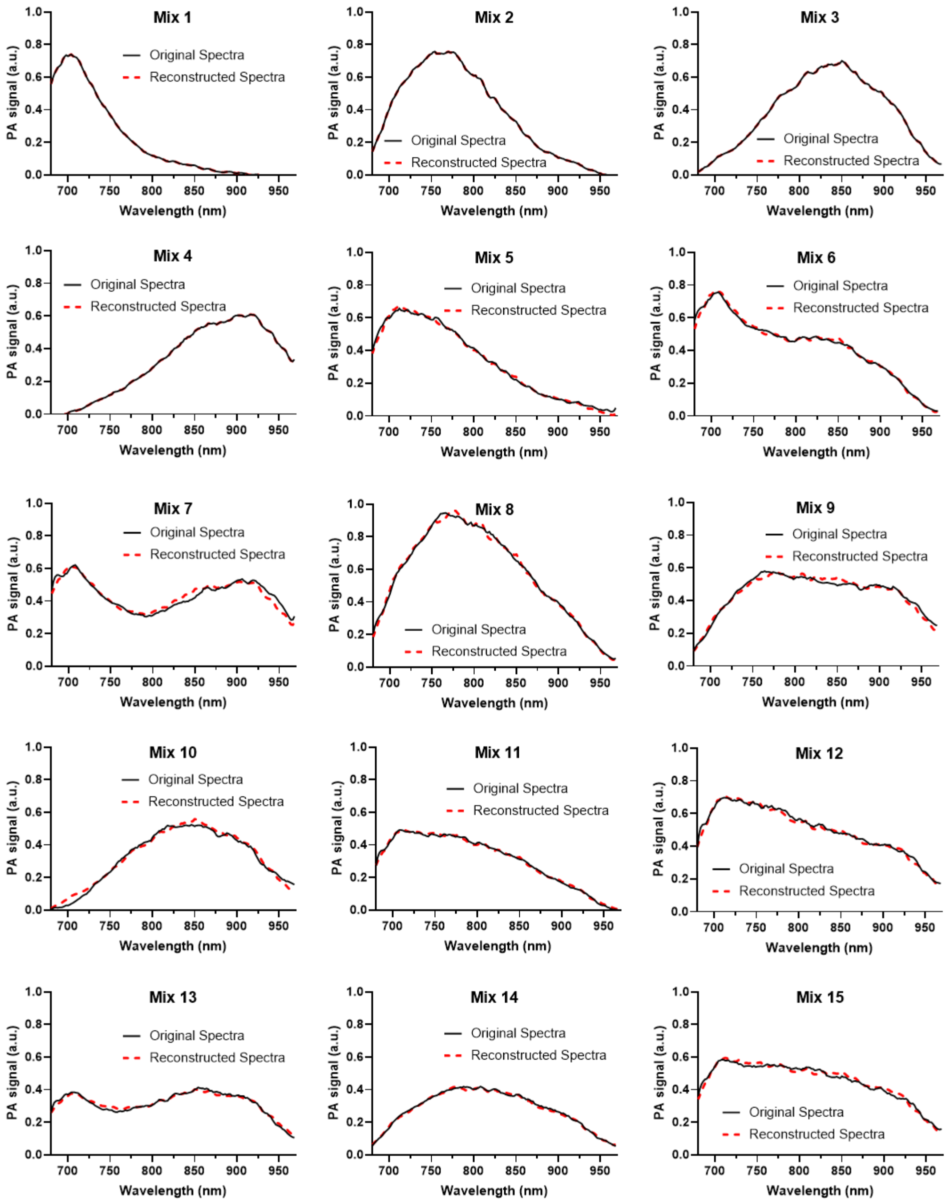

Fig S11. Comparison of original and reconstructed PA spectra for mixtures 1–15. Original versus reconstructed photoacoustic spectra for mixtures 1–15, generated using non-negative least squares (NNLS) spectral unmixing. Original spectra are derived from the known ground truth compositions, while reconstructed spectra use the estimated contributions output of the NNLS multiplexing algorithm. Each plot demonstrates the accuracy of spectral recovery across varying combinations of AuNR1–4.

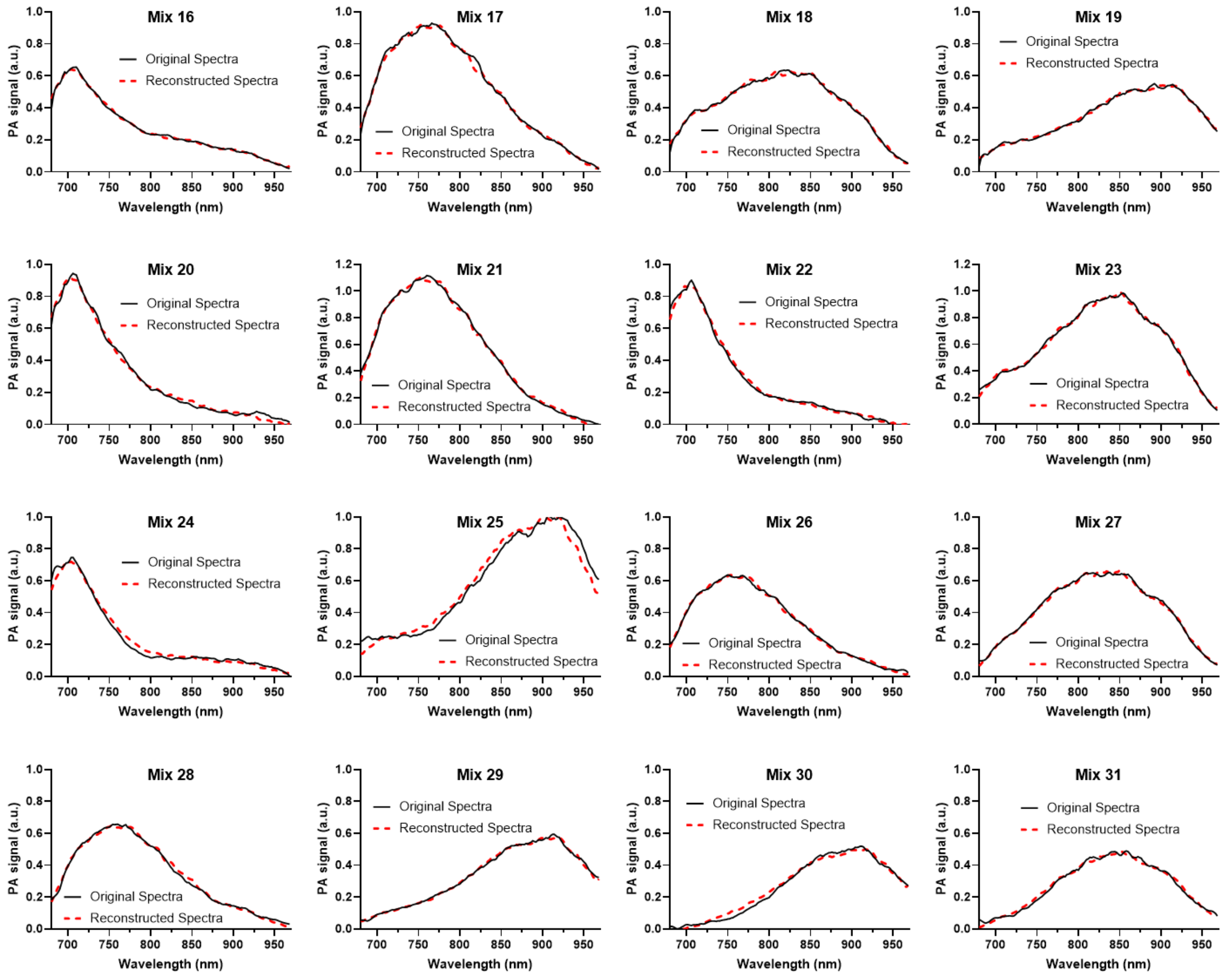

Fig S12. Comparison of original and reconstructed PA spectra for mixtures 16–31. Original versus reconstructed photoacoustic spectra for mixtures 16–31, including boundary binary and quaternary cases. Spectra were unmixed using the NNLS algorithm, with original curves based on ground truth agent composition and reconstructed curves based on estimated contributions. These comparisons validate the algorithm's performance across mixtures with skewed or overlapping spectral profiles.

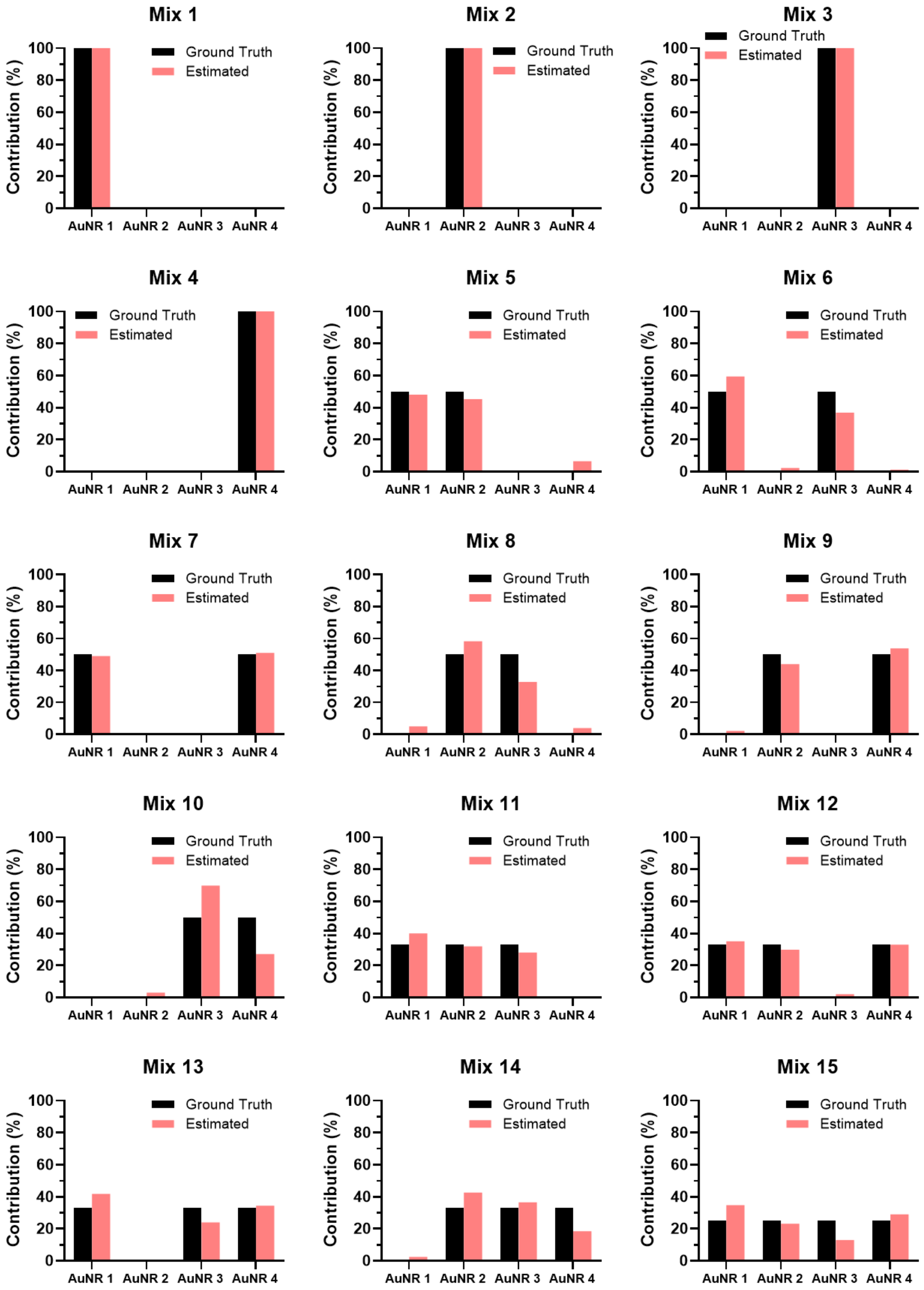

Fig S13. Estimated versus ground truth nanorod contributions for mixtures 1–15. Bar graphs comparing estimated and ground truth contribution percentages of AuNR1–4 for mixtures 1–15. Results are derived from non-negative least squares spectral unmixing. Each group of bars represents one mixture, with estimated and ground truth values shown side by side. The close alignment across most mixtures demonstrates the accuracy of the multiplexing approach.

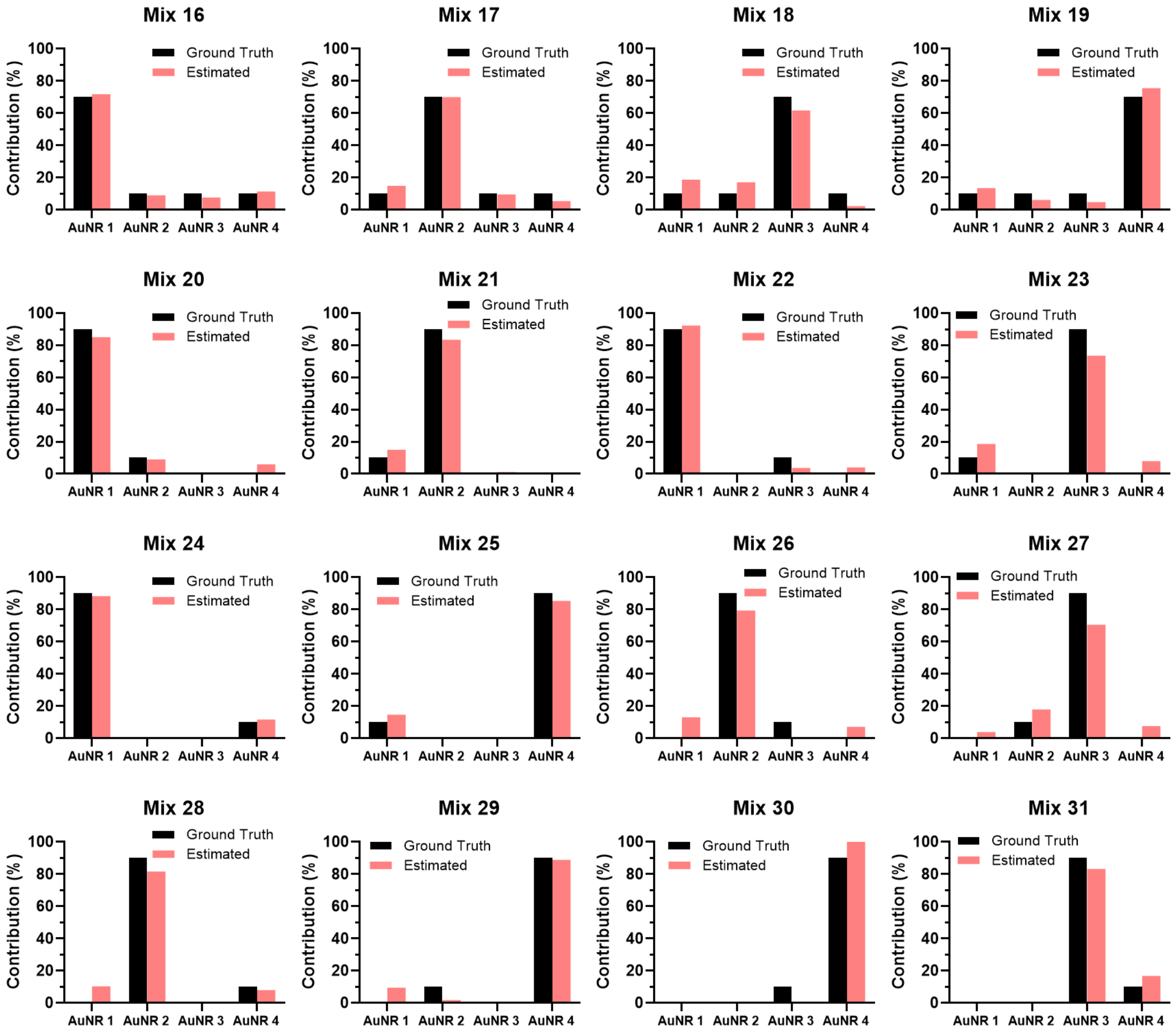

Fig S14. Estimated versus ground truth nanorod contributions for mixtures 16–31. Bar graphs comparing estimated and ground truth contributions for mixtures 16–31, including boundary binary and quaternary cases. Each group shows the estimated versus true percentage for AuNR1–4. Most mixtures exhibit strong agreement, further validating the robustness of the unmixing algorithm in complex or skewed spectral conditions.

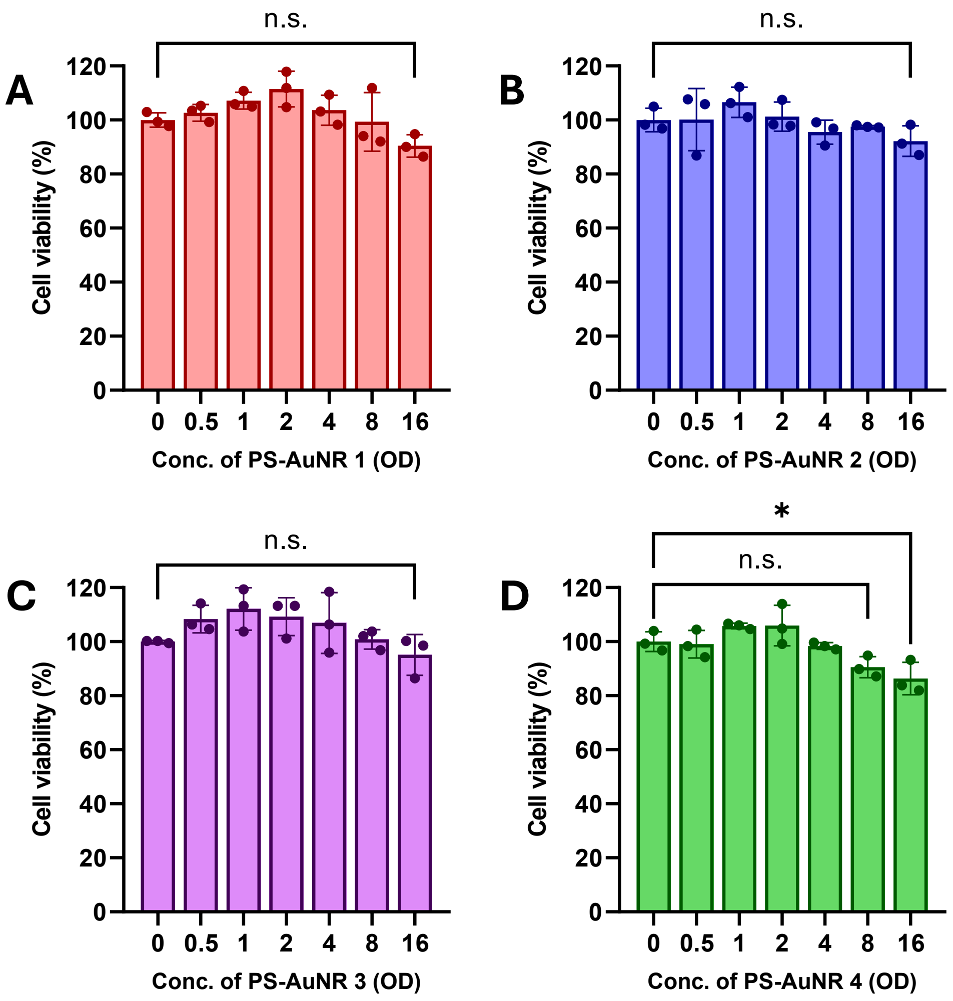

Fig S15. Cytotoxicity assessment of PS-AuNR formulations in hADMSCs. (A–D) Cell viability following incubation with PS-AuNR1, PS-AuNR2, PS-AuNR3, and PS-AuNR4, respectively, over a concentration range of 0, 0.5, 1, 2, 4, 8, and 16 OD. Data are shown as mean ± SD (n = 3 independent replicates). Statistical comparisons were performed relative to the 0 OD control within each panel using one-way ANOVA with Dunnett's multiple-comparisons test. *$p < 0.05$.

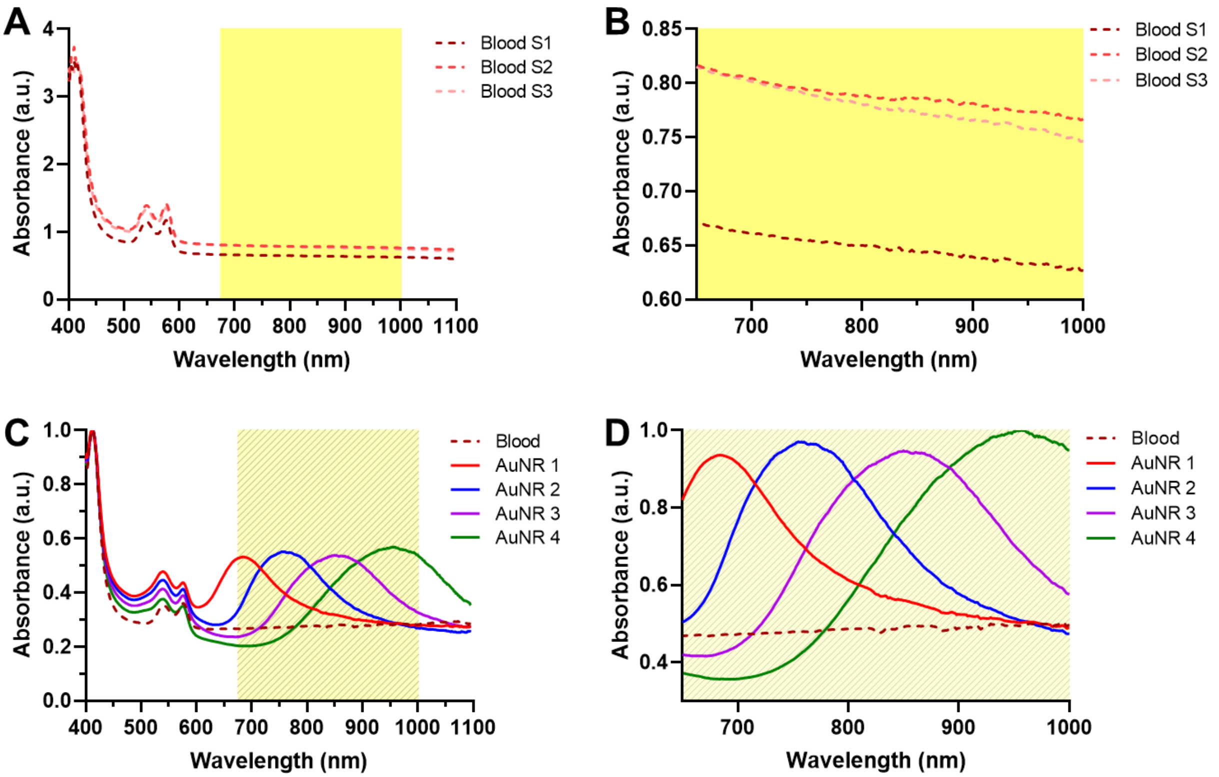

Fig S16. UV-Vis spectral analysis of blood and AuNRs. A. UV-Vis-NIR absorbance spectra of whole blood from three independent samples, highlighting high absorption in the visible region and a sharp decrease in the near-infrared (NIR) region. The NIR-I optical window (~650–1000 nm), used for PA imaging, is indicated by a yellow box. **B.** Zoomed-in view of the NIR-I window from Panel A, showing the absorbance profile of blood across this spectral range. **C.** UV-Vis-NIR spectra of AuNR1–4 mixed with blood, demonstrating that each formulation retains its distinct longitudinal plasmon resonance peak in the NIR region. The nanorods exhibit significantly higher absorbance than blood in this range. **D.** Zoomed-in view of Panel C within the NIR-I window, showing clear spectral separation of the four AuNRs from one another and from the whole-blood background, supporting their suitability for multiplexed PA imaging in the presence of endogenous hemoglobin absorbers.

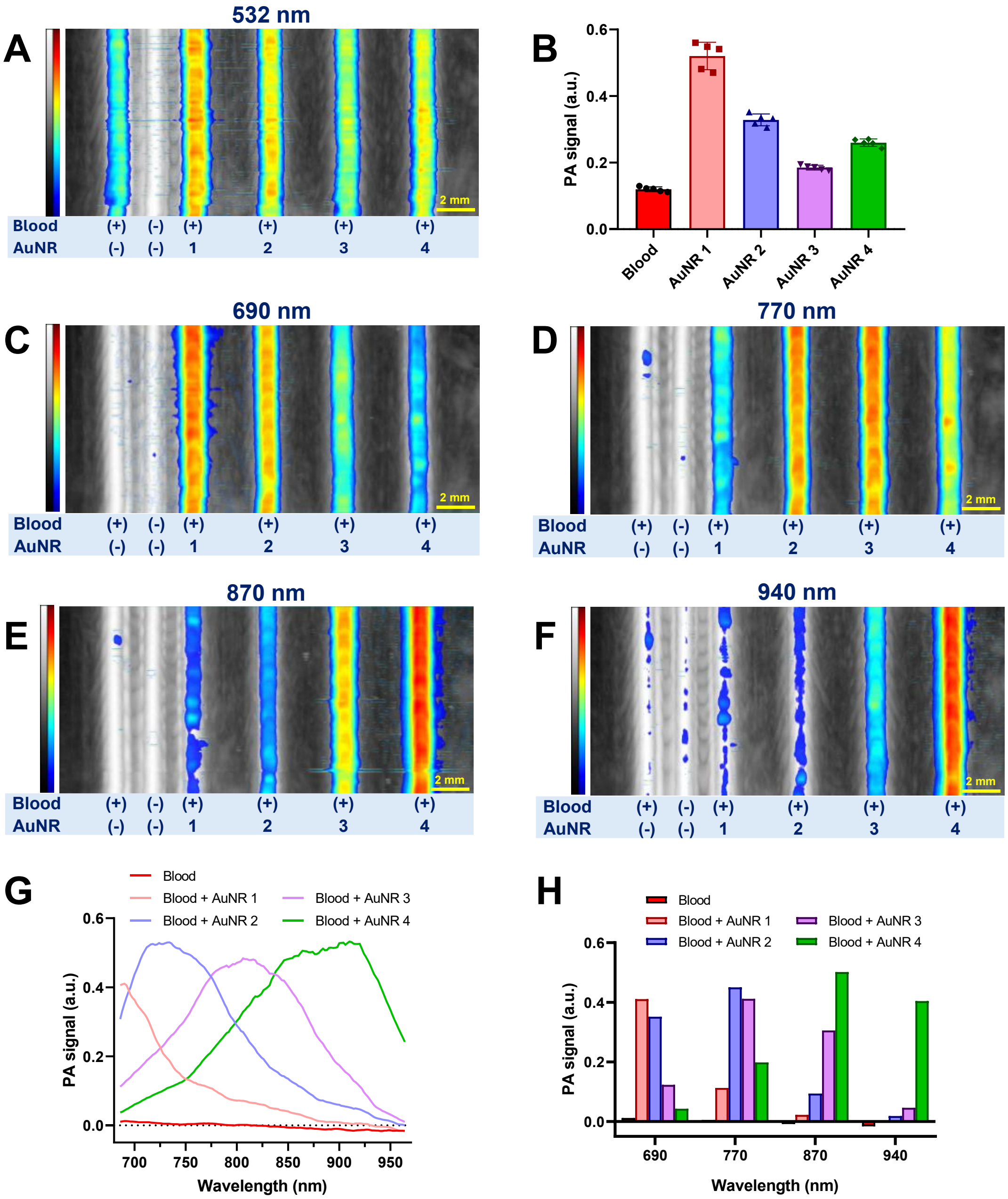

Fig S17. Photoacoustic imaging of AuNRs in blood to assess spectral performance in a physiologically relevant background. A. Representative US/PA image of a tube phantom containing DI water, blood alone, and blood mixed with AuNR1–4, acquired at 532 nm. **B.** Quantified PA signal intensities corresponding to the conditions shown in Panel A. **C–F.** US/PA images of the same phantom acquired at 690 nm (C), 770 nm (D), 870 nm (E), and 940 nm (F), representing the peak absorption wavelengths of AuNR1–4, respectively. **G.** PA spectra of blood and AuNR1–4 in blood across the NIR window (680-970nm), showing that each nanorod maintains a distinct spectral profile above the blood background. Because whole blood contains both oxyhemoglobin and deoxyhemoglobin, this experiment provides a physiologically relevant assessment of endogenous spectral interference. **H.** Bar graph of PA signal intensity for each sample (blood and AuNR1–4 in blood) at the four imaging wavelengths used in Panels C–F, highlighting wavelength-specific enhancement and contrast.

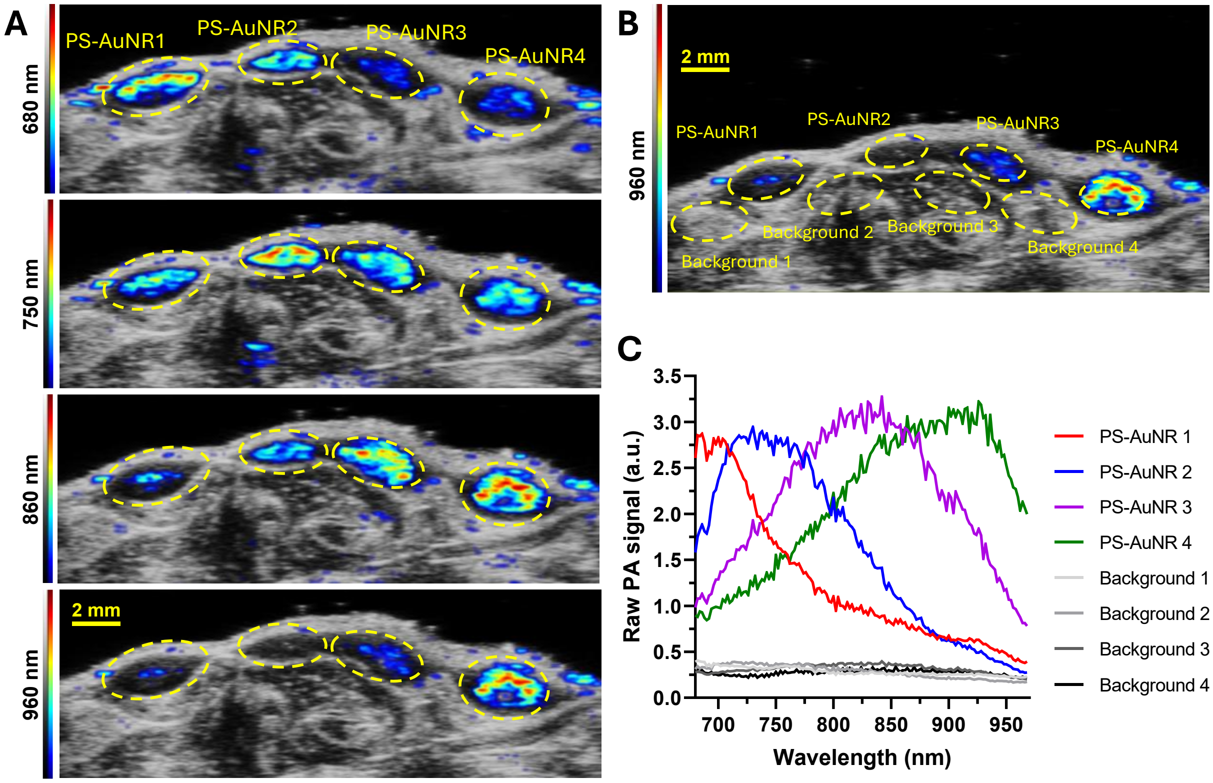

Fig S18. Raw *in vivo* PA background signal across the NIR-I window. (A) Representative *in vivo* US/PA images acquired at 680, 750, 860, and 960 nm of the single agent injections experimental group. (B) Same representative image at 960 nm showing ROIs drawn over the four PS-AuNR injection boluses and four new same-sized background ROIs placed in nearby tissue at comparable depths. ROIs are depicted with dashed yellow lines. (C) Raw PA spectra extracted from the four PS-AuNR ROIs and four background ROIs across the acquisition range, with no image processing, smoothing, or filtering. Endogenous background signals remained low across the NIR-I window relative to the PS-AuNR boluses, supporting robust *in vivo* multiplexing.

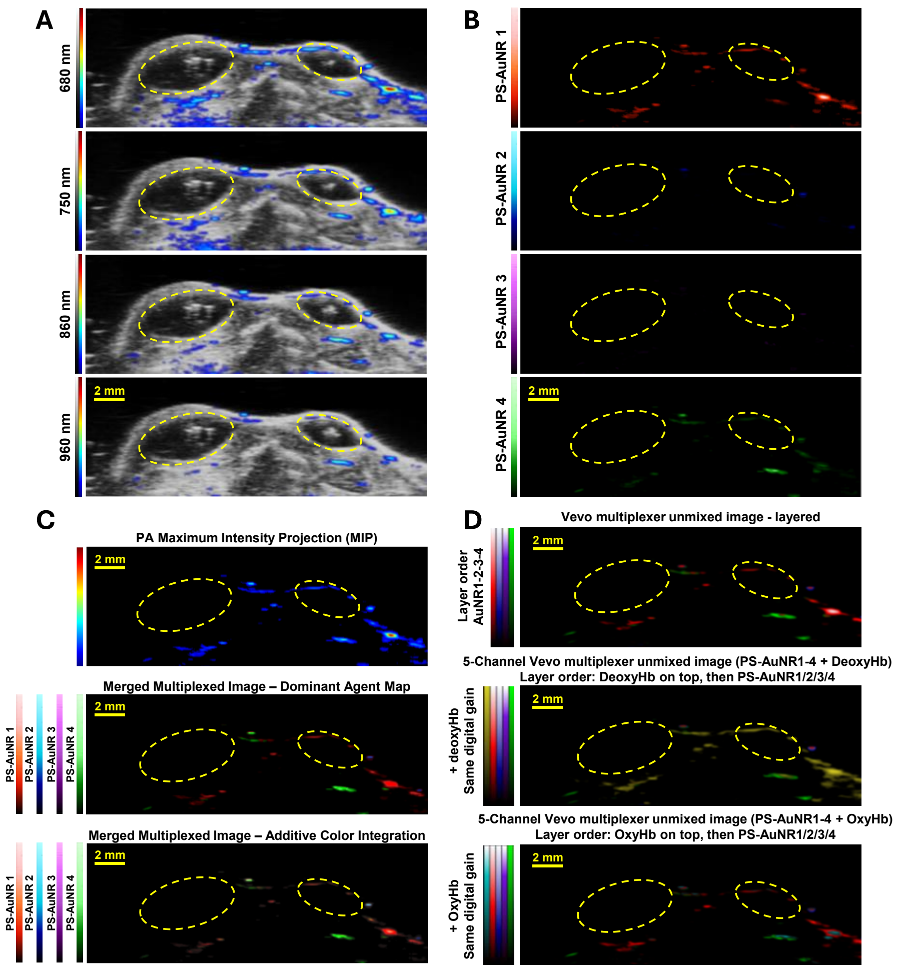

Fig S19. Negative control evaluation of subcutaneous injections with Matrigel-only and PBS only formulations. A. US/PA images acquired at 680, 750, 860, and 960 nm following subcutaneous injection of PBS mixed with Matrigel (left) and PBS alone (right). Injection sites are outlined with dashed yellow lines. No significant PA signal is detected in either region. **B.** Multiplexed single-agent contribution maps for PS-AuNR1–4 in the same regions. No signal is detected from any of the four nanorods within the dashed outlines. Slight residual signal at shorter wavelengths (e.g., 680 nm) is attributed to endogenous absorbers and may produce minor false-positive mapping in PS-AuNR1 because of partial spectral similarity. **C.** Top: PA MIP image showing no signal at the injection sites. Middle: dominant-agent multiplexed map showing only minor background prediction, primarily in the PS-AuNR1 and PS-AuNR4 channels. Bottom: additive merged multiplexed image confirming the absence of detectable PA signal or nanorod contribution at the injection sites. Together, these data support the specificity of the spectral unmixing pipeline under in vivo conditions. **D.** Top: four-channel unmixed image generated using the native VevoLAB spectral deconvolution workflow. Middle: VevoLAB rendering after addition of a deoxyhemoglobin channel. Bottom: VevoLAB rendering after addition of an oxyhemoglobin channel. Because VevoLAB supports a maximum of five channels and renders channels as sequential overlays, deoxyhemoglobin or oxyhemoglobin was placed at the top of the display stack for visibility, followed by PS-AuNR1, PS-AuNR2, PS-AuNR3, and PS-AuNR4 in fixed order. PS-AuNR1, PS-AuNR2, PS-AuNR3, and PS-AuNR4 are shown in red, blue, purple, and green, respectively; deoxyhemoglobin is shown in yellow, and oxyhemoglobin is shown in teal.

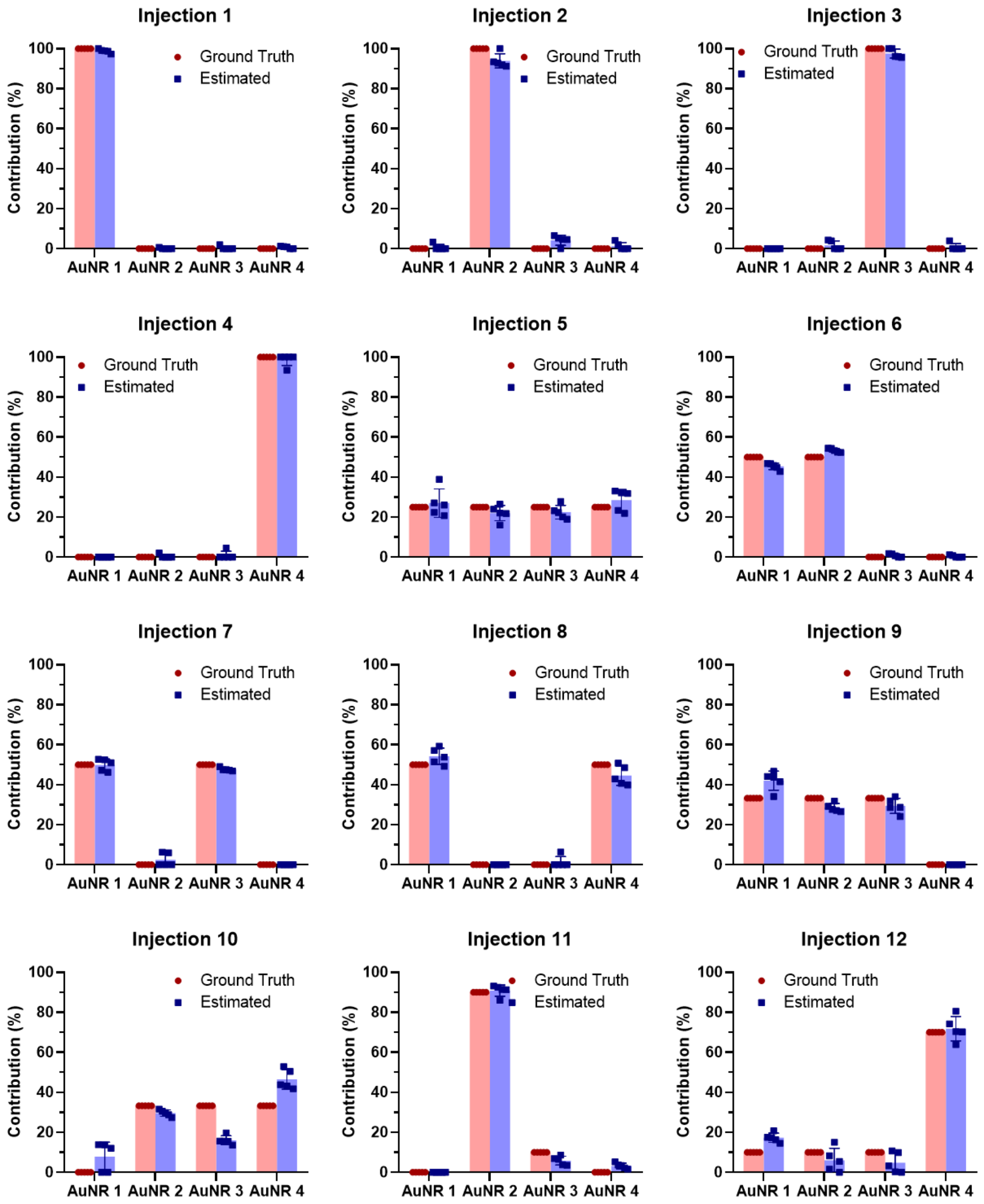

Fig S20. Estimated versus ground truth nanorod contributions for subcutaneous injections (Groups 1–12). Bar graphs comparing estimated and ground truth contribution percentages of AuNR1–4 for all 12 in vivo injection groups (n = 5 per group), based on NNLS spectral unmixing. Each bar shows mean ± standard deviation across biological replicates along with symbols for each replicate. Strong agreement is observed in most cases, validating multiplexing performance under *in vivo* conditions.

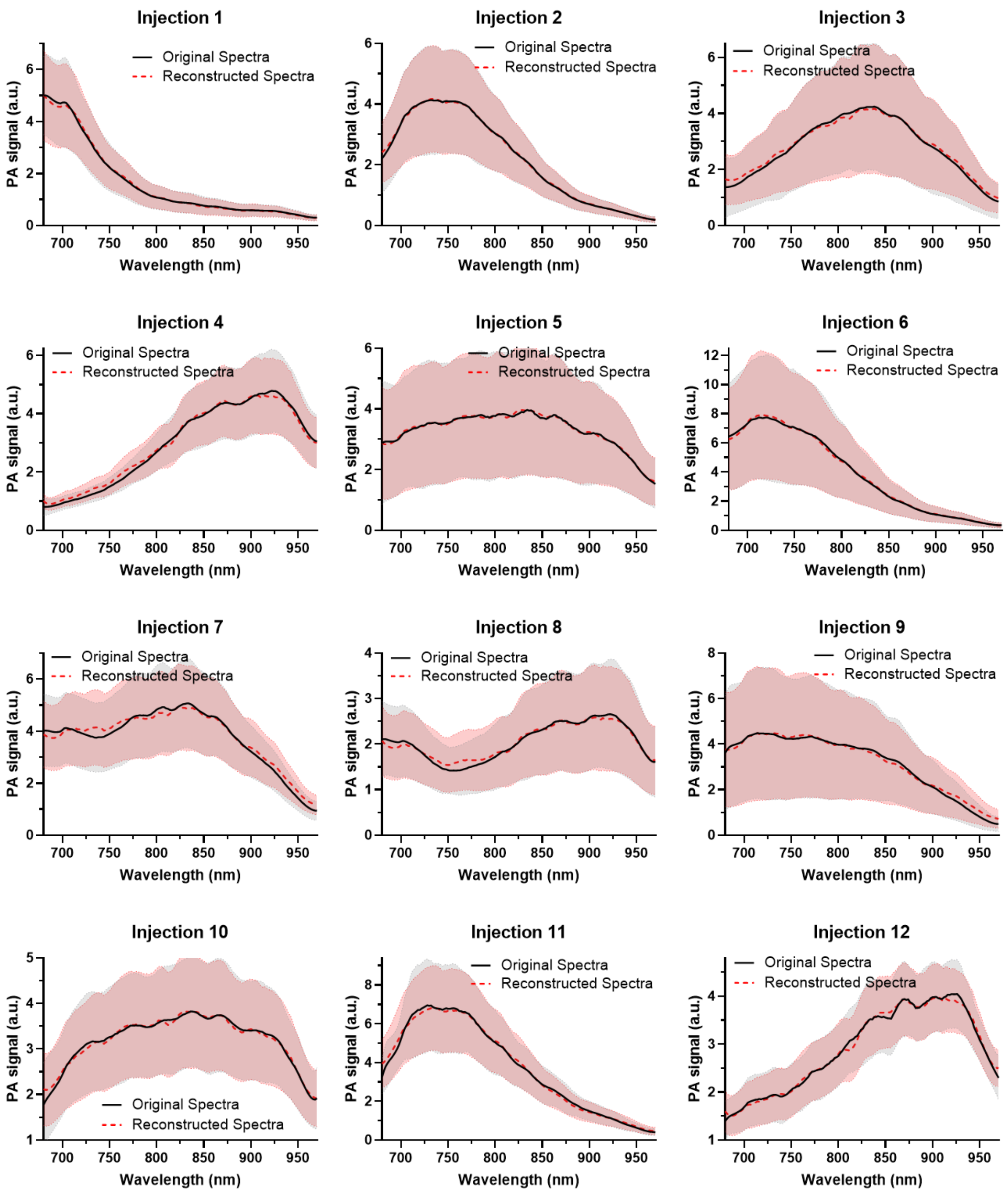

Fig S21. Comparison of original and reconstructed PA spectra for subcutaneous injections (Groups 1–12). Estimated versus original PA spectra for all 12 in vivo injection groups (n = 5 per group), generated using NNLS spectral multiplexing. Original spectra are derived from ground truth mixture compositions, while reconstructed spectra are based on estimated contribution multiplexing outputs. Each graph includes error bars representing standard deviation across replicates, demonstrating robust spectral recovery across single, binary, ternary, quaternary, and boundary conditions.

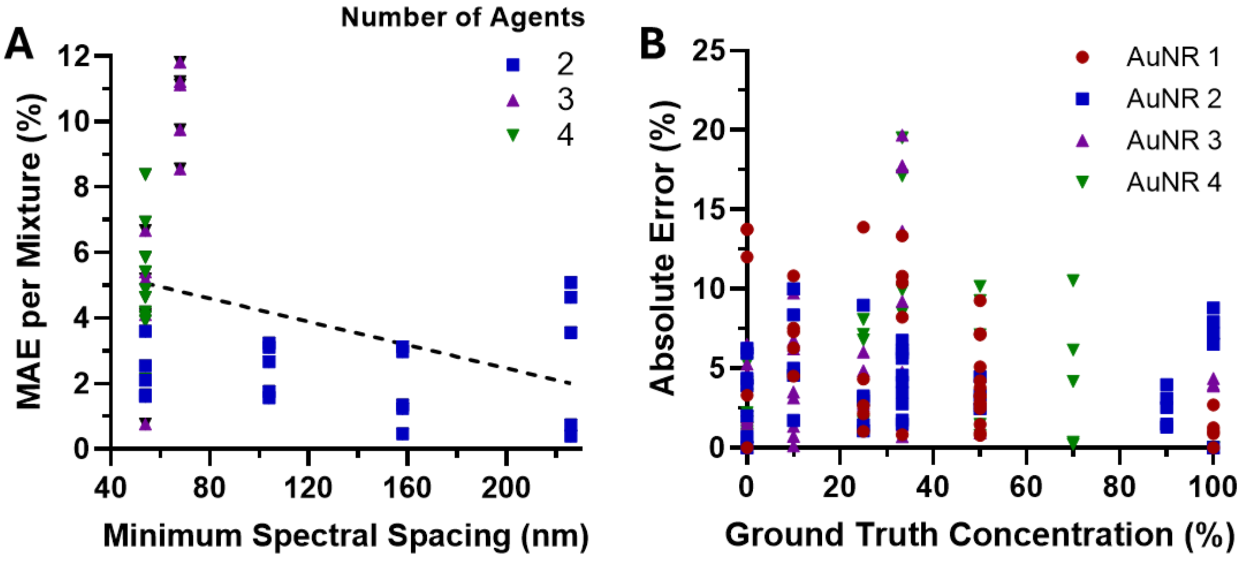

Fig S22. Analysis of spectral spacing and agent concentration effects on unmixing accuracy in subcutaneous injections. A. Mean absolute error (MAE, in %) per injection group plotted against the minimum spectral spacing between AuNRs in each mixture, showing a clear inverse relationship. Greater spectral spacing leads to lower unmixing error. **B.** Absolute error (%) for each contrast agent across all mixtures as a function of its ground truth contribution. No consistent trend is observed, indicating that unmixing error is not strongly dependent on the agent's concentration within a mixture.

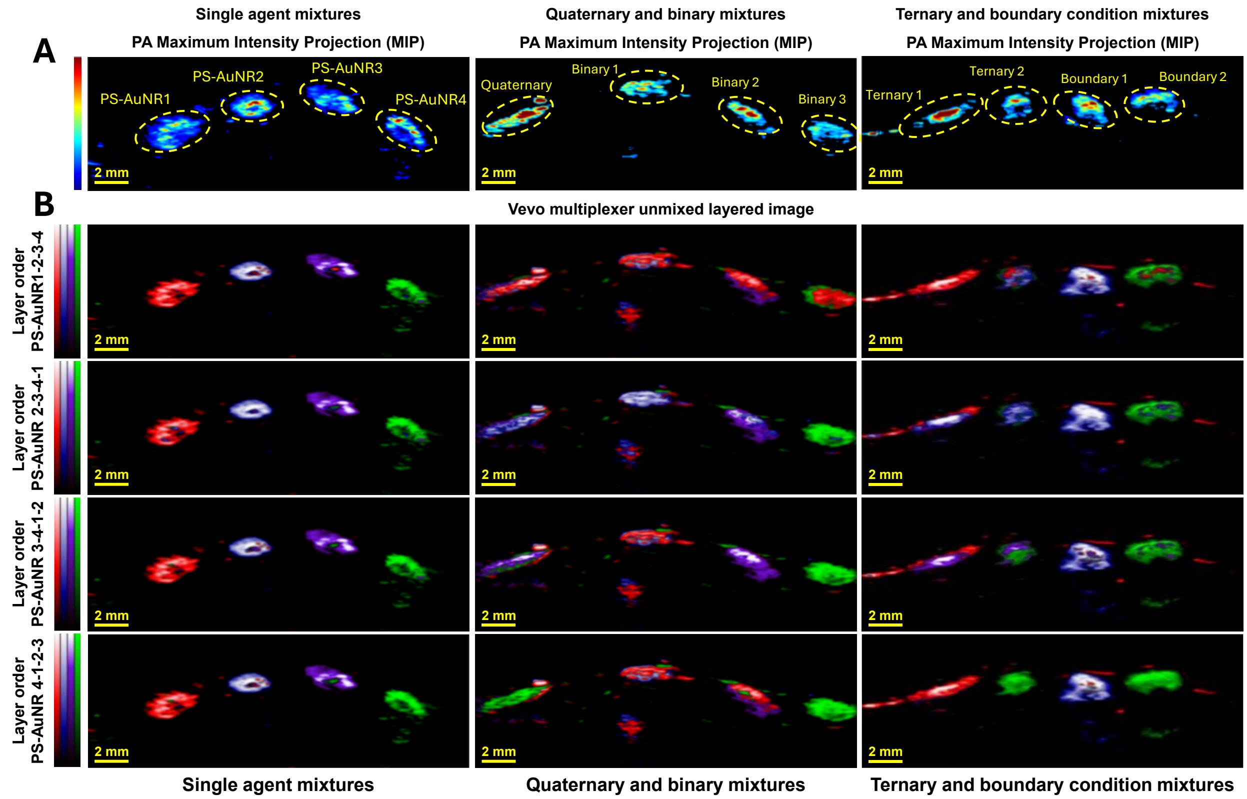

Fig S23. Effect of layer order on built-in VevoLAB unmixed images. A. PA MIP images for the experimental groups: Column 1: Single agent mixtures, Column 2: Quaternary and binary mixtures, and Column 3: Ternary and boundary condition mixtures. **B.** Representative *in vivo* subcutaneous multiplexing datasets from are shown using the built-in VevoLAB spectral unmixing workflow with different fixed layer orders. Four different layer orders shown for the same three columns above, Row 1: PS-AuNR 1-2-3-4, Row 2: PS-AuNR 2-3-4-1, Row 3: PS-AuNR 3-4-1-2, and Row 4: PS-AuNR 4-1-2-3. Agent colors were held constant across all renderings, with PS-AuNR1 shown in red, PS-AuNR2 in blue, PS-AuNR3 in purple, and PS-AuNR4 in green. Because VevoLAB renders channels sequentially as layered overlays, the apparent prominence of a given channel depends in part on display order rather than solely on the recovered signal contribution. These renderings show that the overall multiplexing pattern remains broadly consistent across layer orders, while the visual prominence of individual channels varies depending on which layer is placed on top.

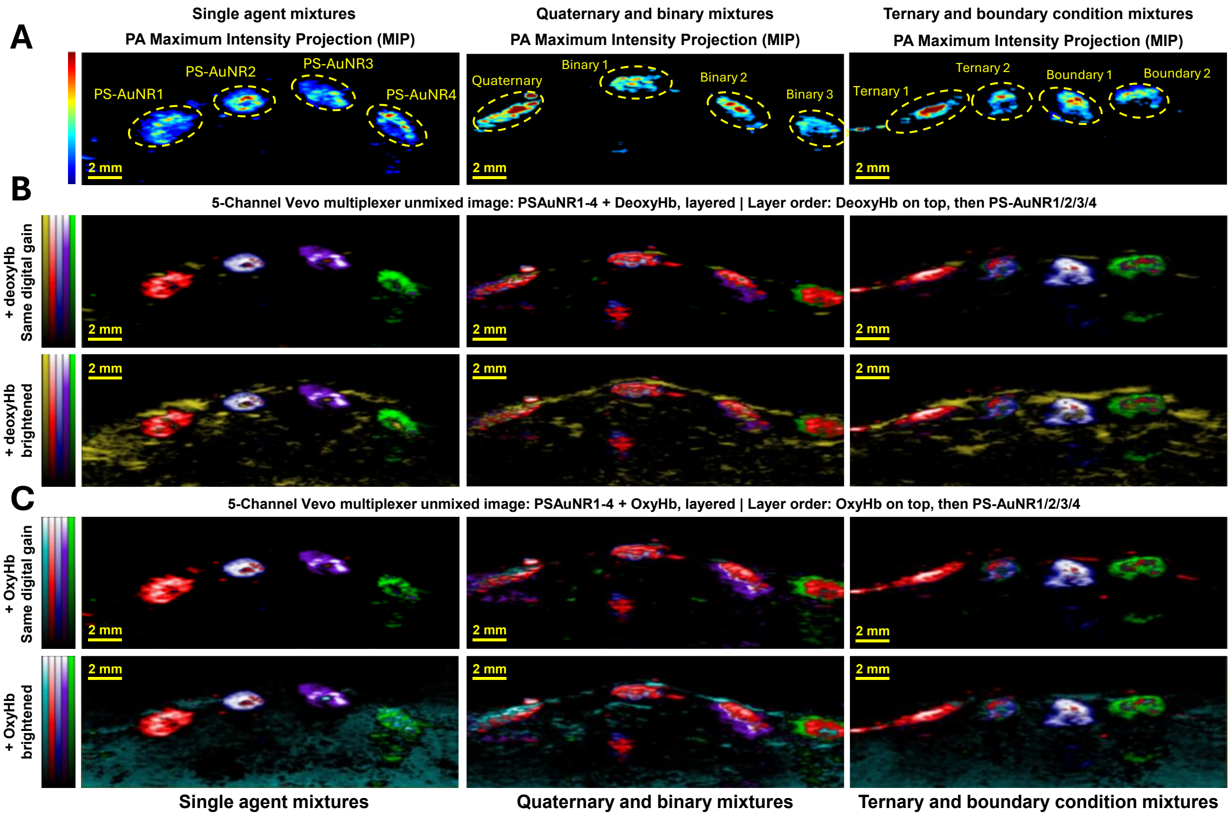

Fig S24. ***In vivo* spectral unmixing of PS-AuNRs with added endogenous hemoglobin basis spectra.** Representative *in vivo* subcutaneous multiplexing datasets: single-agent mixtures (column 1), quaternary and binary mixtures (column 2), and ternary and boundary-condition mixtures (column 3). **A.** PA maximum-intensity-projection (PA MIP) images. **B.** VevoLAB multiplexed renderings after addition of a deoxyhemoglobin channel. The top row shows all the channels displayed under the same visualization settings, while the bottom row shows substantially increased brightness applied only to the deoxyhemoglobin channel to visualize low-level endogenous signal. **C.** VevoLAB multiplexed renderings after addition of an oxyhemoglobin channel. The top row shows all the channels displayed under the same visualization settings, while the bottom row shows substantially increased brightness applied only to the oxyhemoglobin channel. Because the built-in VevoLAB spectral unmixing workflow has a maximum capacity of five channels and renders channels as sequential overlays, deoxyhemoglobin or oxyhemoglobin was placed at the top of the display stack for visibility, followed by PS-AuNR1, PS-AuNR2, PS-AuNR3, and PS-AuNR4 in fixed order. Even under exaggerated brightness conditions, endogenous hemoglobin contributions remained weak relative to the PS-AuNR boluses and did not materially interfere with nanoparticle localization. PS-AuNR1, PS-AuNR2, PS-AuNR3, and PS-AuNR4 are shown in red, blue, purple, and green, respectively; deoxyhemoglobin is shown in yellow, and oxyhemoglobin is shown in teal.

Table S1. Longitudinal LSPR peak FWHM values for AuNR1–4 before and after silica coating

	AuNR	PS-AuNR	Increase in FWHM
1	75.31 nm	101.65 nm	26.34 nm
2	85.46 nm	130.17 nm	44.71 nm
3	115.21 nm	174.74 nm	59.53 nm
4	118.89 nm	189.19 nm	70.30 nm

Table S2. Comparison of longitudinal peak FWHM values from measured absorbance and corresponding photoacoustic spectra for PS-AuNR1–4.

Probe	Absorbance FWHM	PA FWHM	PA – Absorbance
PS-AuNR1	101.65 nm	83.93 nm	-17.72 nm
PS-AuNR2	130.17 nm	144.21 nm	14.04 nm
PS-AuNR3	174.74 nm	143.98 nm	-30.76 nm
PS-AuNR4	189.19 nm	199.33 nm	10.14 nm

Table S3. Ground truth nanorod contributions for *in vitro* multiplexing experiments.

Mixture No.	PS-AuNR 1	PS-AuNR 2	PS-AuNR 3	PS-AuNR 4
1	100	0	0	0
2	0	100	0	0
3	0	0	100	0
4	0	0	0	100
5	50	50	0	0
6	50	0	50	0
7	50	0	0	50
8	0	50	50	0
9	0	50	0	50
10	0	0	50	50
11	33.33	33.33	33.33	0
12	33.33	33.33	0	33.33
13	33.33	0	33.33	33.33
14	0	33.33	33.33	33.33
15	25	25	25	25
16	70	10	10	10
17	10	70	10	10
18	10	10	70	10
19	10	10	10	70
20	90	10	0	0
21	10	90	0	0
22	90	0	10	0
23	10	0	90	0
24	90	0	0	10
25	10	0	0	90
26	0	90	10	0
27	0	10	90	0
28	0	90	0	10
29	0	10	0	90
30	0	0	10	90
31	0	0	90	10

Table S4. Estimated nanorod contributions for *in vitro* mixtures after NNLS spectral unmixing.

Mixture No.	PS-AuNR 1	PS-AuNR 2	PS-AuNR 3	PS-AuNR 4
1	100	0	0	0
2	0	100	0	0
3	0	0	100	0
4	0	0	0	100
5	48.19	45.29	0	6.53
6	59.5	2.33	36.91	1.27
7	49.03	0	0	50.97
8	4.9	58.27	32.81	4.02
9	2.16	44.03	0	53.82
10	0	3.03	69.88	27.09
11	40.02	31.89	28.08	0
12	35.11	29.86	2.08	32.96
13	41.76	0	23.9	34.34
14	2.34	42.65	36.49	18.53
15	34.74	23.18	13.05	29.03
16	71.85	9.03	7.65	11.46
17	14.91	70.05	9.64	5.4
18	18.72	17.18	61.78	2.32
19	13.58	6.14	4.7	75.58
20	85.08	9	0	5.92
21	14.97	83.49	1.16	0.39
22	92.33	0	3.65	4.02
23	18.58	0	73.53	7.88
24	88.28	0	0	11.72
25	14.71	0	0	85.29
26	13.1	79.33	0.39	7.18
27	3.87	17.95	70.51	7.66
28	10.4	81.59	0	8.01
29	9.46	1.78	0	88.76
30	0	0	0	100
31	0	0	83.17	16.83

Table S5. Ground truth nanorod contributions for *in vivo* subcutaneous multiplexing experiments.

Mixture	PS-AuNR 1	PS-AuNR 2	PS-AuNR 3	PS-AuNR 4	Measurement
1	100	0	0	0	1-5
2	0	100	0	0	6-10
3	0	0	100	0	11-15
4	0	0	0	100	16-20
5	25	25	25	25	21-25
6	50	50	0	0	25-30
7	50	0	50	0	31-35
8	50	0	0	50	35-40
9	33.33	33.33	33.33	0	41-45
10	0	33.33	33.33	33.33	45-50
11	0	90	10	0	51-55
12	10	10	10	70	55-60

Table S6. Estimated nanorod contributions for in vivo subcutaneous mixtures after spectral unmixing.

Measurement	Experimental Group	PS-AuNR 1	PS-AuNR 2	PS-AuNR 3	PS-AuNR 4
1	1	98.97	0	0	1.03
2	1	100	0	0	0
3	1	98.73	0	0	1.27
4	1	99.11	0	0	0.89
5	1	97.31	0.7	1.93	0.06
6	2	3.3	92.08	4.63	0
7	2	0	100	0	0
8	2	0	91.2	4.67	4.13
9	2	0	92.81	5.25	1.94
10	2	0	93.49	6.51	0
11	3	0	3.89	96.11	0
12	3	0	0	96.02	3.98
13	3	0	0	100	0
14	3	0	0	100	0
15	3	0	4.36	95.64	0
16	4	0	0	0	100
17	4	0	0	0	100
18	4	0	2.02	4.51	93.47
19	4	0	0	0	100
20	4	0	0	0	100
21	5	26.04	22.01	20.15	31.79
22	5	38.88	16.04	23.21	21.88
23	5	20.68	23.95	22.3	33.08
24	5	27.14	21.75	18.97	32.14
25	5	22.35	26.44	27.82	23.39
26	6	42.82	54.46	1.53	1.19
27	6	45.78	52.47	1.75	0
28	6	46.7	53.3	0	0
29	6	44.91	54.38	0.71	0
30	6	46.79	52.39	0	0.82
31	7	50.93	0	49.07	0
32	7	47.17	5.94	46.89	0
33	7	46.22	6.25	47.53	0

34	7	52.46	0	47.54	0
35	7	52.68	0	47.32	0
36	8	57.11	0	0	42.89
37	8	51.47	0	0	48.53
38	8	59.26	0	0	40.74
39	8	49.22	0	0	50.78
40	8	53.82	0	6.34	39.84
41	9	41.55	26.56	31.89	0
42	9	44.14	27.16	28.7	0
43	9	43.72	27.7	28.58	0
44	9	34.16	31.81	34.03	0
45	9	46.67	29.2	24.12	0
46	10	0	29.85	19.7	50.45
47	10	12.01	30.56	13.65	43.78
48	10	13.77	28.76	15.65	41.82
49	10	0	31.59	15.56	52.85
50	10	13.73	27.37	15.66	43.23
51	11	0	91.49	6.87	1.64
52	11	0	93.12	3.54	3.34
53	11	0	92.54	3.79	3.67
54	11	0	91.31	6.5	2.19
55	11	0	86.03	8.65	5.32
56	12	14.51	15.01	0.26	70.22
57	12	17.3	1.63	10.72	70.34
58	12	17.53	8.29	0	74.18
59	12	16.28	0	3.23	80.5
60	12	20.82	5.45	9.88	63.85
